# A tumor-targeted heptamethine cyanine dye suppresses triple-negative breast cancer by induction of lethal autophagy

**DOI:** 10.7150/thno.130353

**Published:** 2026-04-23

**Authors:** Sang-Hyo Kim, Yoonbin Park, Hwa-yeong Jin, Taewon Lee, Sungsu Lee, Moon Suk Kim, Hoon Hyun

**Affiliations:** 1 Department of Biomedical Sciences, Chonnam National University Medical School, Hwasun 58128, South Korea.; 2 BioMedical Sciences Graduate Program (BMSGP), Chonnam National University, Hwasun 58128, South Korea.; 3 Division of Applied Mathematical Sciences, College of Science and Technology, Korea University, Sejong 30019, South Korea.; 4 Department of Otolaryngology-Head and Neck Surgery, Chonnam National University Medical School, Gwangju 61469, South Korea.; 5 Department of Molecular Science and Technology, Ajou University, Suwon 16499, South Korea.

**Keywords:** triple-negative breast cancer, heptamethine cyanine dyes, ESCRT, autophagy, antitumor immunity

## Abstract

**Background:**

Triple-negative breast cancer (TNBC) is a highly invasive type of breast cancers that is characterized by the absence of hormone receptors and HER2 protein, thereby relying mostly on surgical intervention and cytotoxic chemotherapy. Recently, autophagy in TNBC progression has emerged as an important role for more effective TNBC treatments.

**Methods:**

Since autophagy is a critical determinant of cell fate, depending on the context and stress level, we newly develop a hydrophilic anionic heptamethine cyanine dye (named TNBC800) for the treatment of TNBC by induction of lethal autophagy.

**Results:**

TNBC800 induces autophagy-mediated immunogenic cell death to exert targeted therapeutic effects on MDA-MB-231 xenografts. In terms of molecular mechanism, the TNBC800 can be imported into MDA-MB-231 cells through the endosomal sorting complex required for transport (ESCRT) pathway. Consequently, TNBC800 elevates the intracellular level of reactive oxygen species (ROS) and induces autophagic stress, demonstrated by increased LC3B accumulation, which contributes to cell apoptosis and suppression of tumor proliferation. Finally, we confirm a substantial increase in the presence of M1 macrophages in spleen and NK cells in tumors over the course of treatment.

**Conclusion:**

This study introduces a potentially effective strategy for enhancing TNBC treatment efficacy.

## Introduction

Triple-negative breast cancer (TNBC) is clinically characterized by the absence of three key receptors for estrogen, progesterone, and HER2, thereby restricting the availability of targeted therapeutic options. Despite comprising a relatively modest subset of total breast cancer cases (15-20%), TNBC disproportionately contributes to poor clinical outcomes due to its high rates of relapse and metastatic progression 1. Current clinical management primarily relies on cytotoxic chemotherapy in combination with surgical intervention, particularly in early-stage disease, where neoadjuvant or adjuvant regimens are employed to reduce recurrence risk 2,3. However, even with standard agents such as anthracyclines and taxanes, treatment outcomes remain suboptimal in comparison with other breast cancer variants, highlighting the limitations of existing therapeutic strategies 4. Although emerging targeted approaches, including bromodomain and extra-terminal domain inhibitors, have shown initial promise, their clinical benefit is often undermined by the rapid development of drug resistance 5. These challenges underscore the pressing need for alternative therapeutic paradigms in TNBC.

Autophagy has been described as a double-edged sword in cancer biology, exerting context-dependent effects that range from cytoprotective functions to the facilitation of cell death. In the therapeutic setting, modulation of autophagy has been explored as a means to sensitize tumor cells or enhance treatment efficacy, leading to the clinical evaluation of both autophagy activators and inhibitors in combination with conventional therapies. In TNBC, accumulating evidence suggests that dysregulation of autophagic processes may be exploited for therapeutic benefit 6-8. In particular, autophagy-dependent cell death (ADCD) has gained attention as a non-apoptotic mechanism in which excessive or sustained autophagic activity drives cellular demise 9-11. Notably, activation of autophagy has also been linked to improved antitumor immunity through enhanced antigen processing and presentation, as well as activation of immune effector cells, suggesting a potential intersection between autophagy and immunogenic cancer therapy 12-14.

In this study, we introduce an innovative small-molecule theranostic agent, TNBC800, a hydrophilic anionic heptamethine cyanine dye designed to preferentially accumulate in TNBC tumors and enable near-infrared (NIR) fluorescence-based imaging. Beyond its imaging capability, TNBC800 exhibited pronounced therapeutic activity in an MDA-MB-231 xenograft model. Mechanistic investigations revealed that treatment with TNBC800 induced a marked increase in intracellular reactive oxygen species (ROS), accompanied by significant accumulation of LC3B, indicative of elevated autophagic flux. This heightened autophagic activity was associated with cellular stress responses that promoted the release of pro-inflammatory cytokines and subsequent elicitation of immunogenic cell death (ICD). Importantly, these effects were coupled with a shift in the tumor immune microenvironment, characterized by increased infiltration of pro-inflammatory M1-like macrophages expressing MHC class II and CD80. Taken together, the current evidence delineates a model in which TNBC800 mediates antitumor efficacy through coordinated activation of autophagy and immune responses, offering a potential therapeutic strategy for TNBC.

## Results and Discussion

### Preparation and characterization of TNBC800

**Figure 1:**
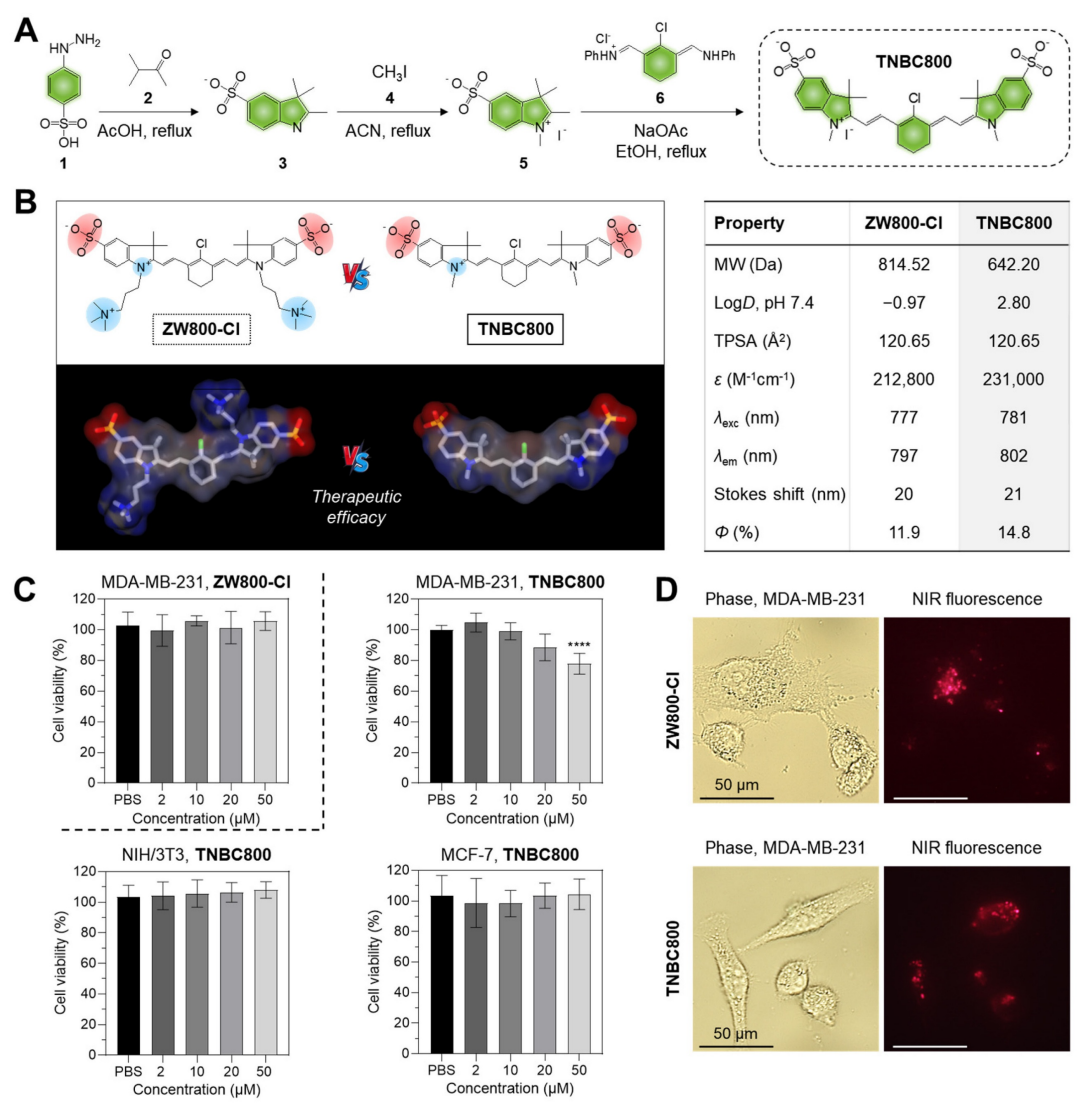
Synthesis, characterization, and cytotoxicity of TNBC800. A) Synthetic route for the water-soluble heptamethine cyanine dye TNBC800. B) 2D, 3D models and properties of TNBC800 or ZW800-Cl. Blue indicates positive charge, red indicates negative charge, and gray represents hydrophobic regions. C) Cytotoxicity of TNBC800 and ZW800-Cl was examined in MDA-MB-231, MCF-7, and NIH/3T3 cell lines. Cytotoxicity was measured after 24 h exposure to increasing concentrations of TNBC800. Values are expressed as mean ± S.D. (^****^p < 0.0001, *n* = 6). D) Cellular binding of TNBC800 or ZW800-Cl was assessed in live MDA-MB-231 cells. All images were acquired at 24 h after incubation with TNBC800 or ZW800-Cl (20 μM). Scale bars = 50 µm.

TNBC800, a hydrophilic anionic NIR small-molecule dye, was constructed from the previously reported hydrophilic zwitterionic heptamethine cyanine dye ZW800-Cl 15,16. Owing to their unique zwitterionic structure, the almost polymethine fluorophores have shown remarkable properties, featuring significant molar absorptivity and optimal solubility in water as established in previous studies, while also demonstrating rapid systemic clearance *in vivo*, which helps reduce nonspecific retention in normal tissues and organs 15. Although ZW800-Cl has been reported to accumulate selectively in tumor xenografts such as NCI-H460, HT-29, and MCF-7, it does not exert direct antitumor effects in these cancer models 17. To endow the dye with both tumor-targeting capability and therapeutic activity, the structure and physicochemical profile of ZW800-Cl were redesigned by modifying the two side chains, including sulfonate and trimethylammonium moieties, according to the previous reports 16,18. In this study, TNBC800 was newly designed as a hydrophilic anionic cyanine dye containing two sulfonate groups and a heptamethine core with a meso-chloride substituent. As illustrated in Figure 1A, TNBC800 was synthesized through a condensation reaction using a mixture of the sulfonated indolium **5**, the Vilsmeier reagent **6**, and anhydrous sodium acetate. Its chemical structure and exact molecular mass were identified by ^1^H-NMR spectroscopy and liquid chromatography-mass spectrometry, respectively, supporting successful synthesis for subsequent cell and animal studies (Figures S1 and S2). Also, TNBC800 was dissolved in phosphate-buffered saline (PBS) to determine the absorption and fluorescence profiles (Figure S3). As shown in Figure 1B, TNBC800 exhibited an increased molar absorption coefficient (231,000 M^-1^cm^-1^) and quantum yield of fluorescence (14.8%), compared to that of ZW800-Cl. Additionally, *in silico* hydrophobicity and polarity of TNBC800 were estimated using JChem software. Although TNBC800 remains water-soluble, it showed a higher log*D* value (2.80) than ZW800-Cl (-0.97), while both compounds had the same topological polar surface area (TPSA; 120.65 Å^2^).

To evaluate whether TNBC800 exhibits cell type-selective toxicity, we first performed viability assays using human breast cancer cells (MDA-MB-231 and MCF-7) and noncancerous NIH/3T3 fibroblasts, and then examined cellular uptake in the MDA-MB-231 TNBC cells. As a result of the MTT colorimetric assay, TNBC800 produced no appreciable toxicity in NIH/3T3 fibroblasts or MCF-7 cells across the concentration range of 2-50 μM (Figure 1C). In contrast, viability of MDA-MB-231 cells decreased in a concentration-dependent manner after TNBC800 exposure, and this reduction was more pronounced than that observed with ZW800-Cl. A similar viability pattern was also observed in 4T1 mouse breast cancer cells, another TNBC model, relative to MDA-MB-231 cells (Figure S4A). These findings suggest that TNBC800 exerts TNBC-selective cytotoxicity and may therefore be useful for both imaging and treatment of TNBC. After establishing its cytotoxic effect in the MDA-MB-231 TNBC cells, we compared the intracellular uptake patterns of TNBC800 or ZW800-Cl following 24 h incubation. ZW800-Cl showed a diffuse and uneven intracellular pattern, whereas TNBC800 exhibited a distinct localization profile resembling lysosomal accumulation (Figure 1D). Under the same conditions, no notable binding or uptake was detected in NIH/3T3 fibroblasts or MCF-7 cells after TNBC800 treatment (Figure S4B). These results imply that the internalization behavior of TNBC800 in MDA-MB-231 cells may underlie its TNBC-selective toxicity.

**Figure 2:**
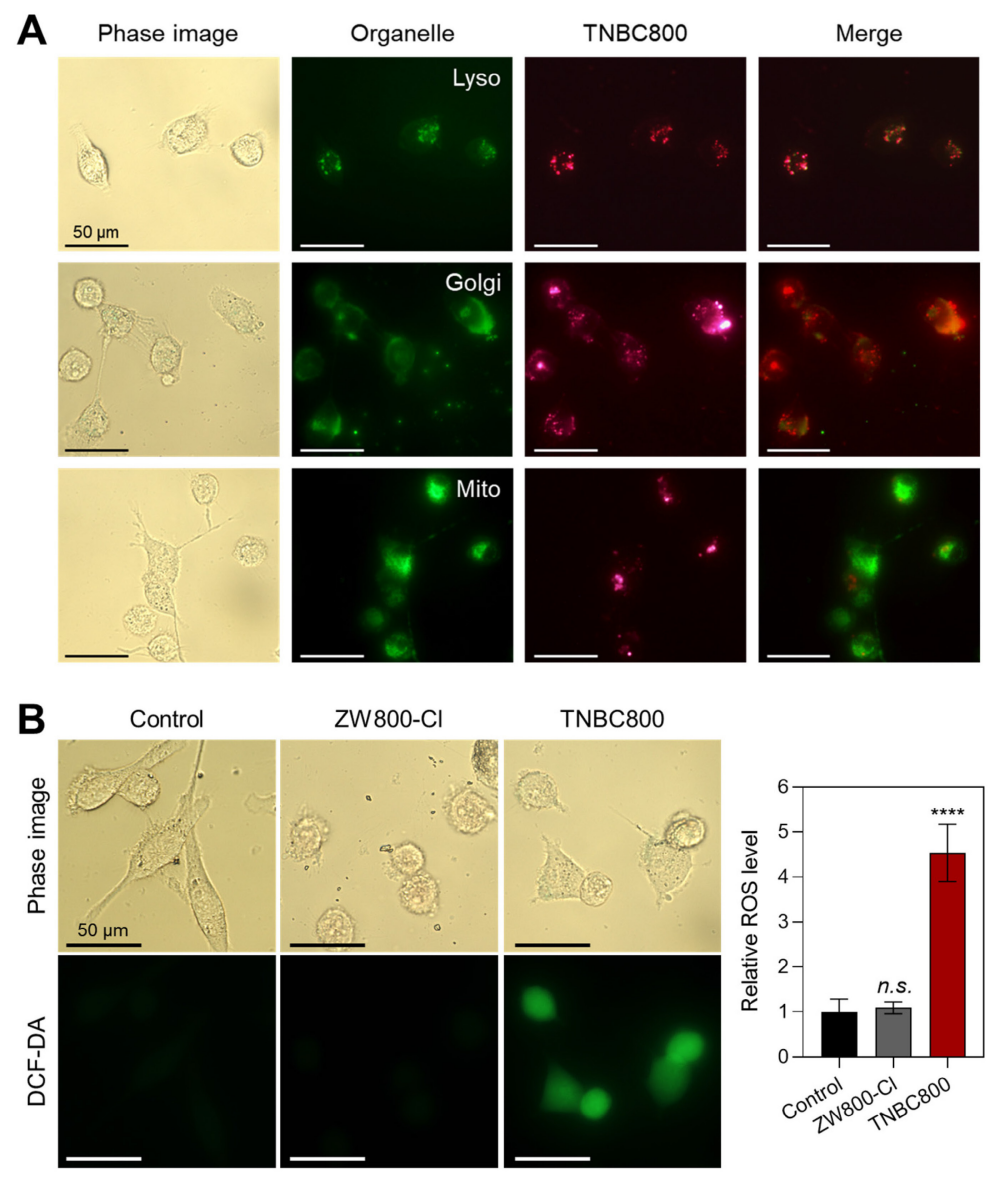
Intracellular localization and ROS generation by TNBC800. A) Live-cell imaging showing the distribution of TNBC800 in MDA-MB-231 cells. TNBC800 was co-labeled with lysosomal, Golgi, and mitochondrial trackers. Microscopy images were captured at 24 h of incubation with 20 μM TNBC800 and subsequent organelle staining. Representative images from three independent experiments are shown. Scale bars = 50 μm. B) Assessment of intracellular ROS production using the DCF-DA assay. Scale bars = 50 μm. All images were acquired with consistent exposure times and normalization across samples. Quantitative results are expressed as mean ± S.D. (^****^p < 0.0001, *n* = 3).

To further identify the subcellular compartment associated with TNBC800, MDA-MB-231 cells were co-stained with Lyso-, Golgi-, and Mito-Trackers after TNBC800 treatment and then examined by fluorescence microscopy (Figure 2A). TNBC800 showed the strongest overlap with Lyso-Tracker, with much weaker co-localization observed for the Golgi and mitochondrial markers. This suggests that TNBC800 may provoke lysosomal stress, a process that could contribute to TNBC-specific apoptosis. Because lysosomal stress is known to participate in programmed cell death and may offer an alternative therapeutic route for drug-resistant tumors 19,20, we next evaluated ROS production in TNBC800-treated MDA-MB-231 cells using a nonfluorescent probe DCF-DA. Notably, TNBC800 induced a strong ROS signal, as shown by bright green fluorescence from oxidized DCF, whereas cells treated with ZW800-Cl displayed fluorescence levels comparable to the untreated control (Figure 2B). Previous studies have shown that elevated ROS can damage lysosomes, disrupt cellular waste processing, and induce autophagic stress, which may ultimately trigger apoptotic cell death 21,22.

### Molecular mechanism of cell death induced by TNBC800

**Figure 3:**
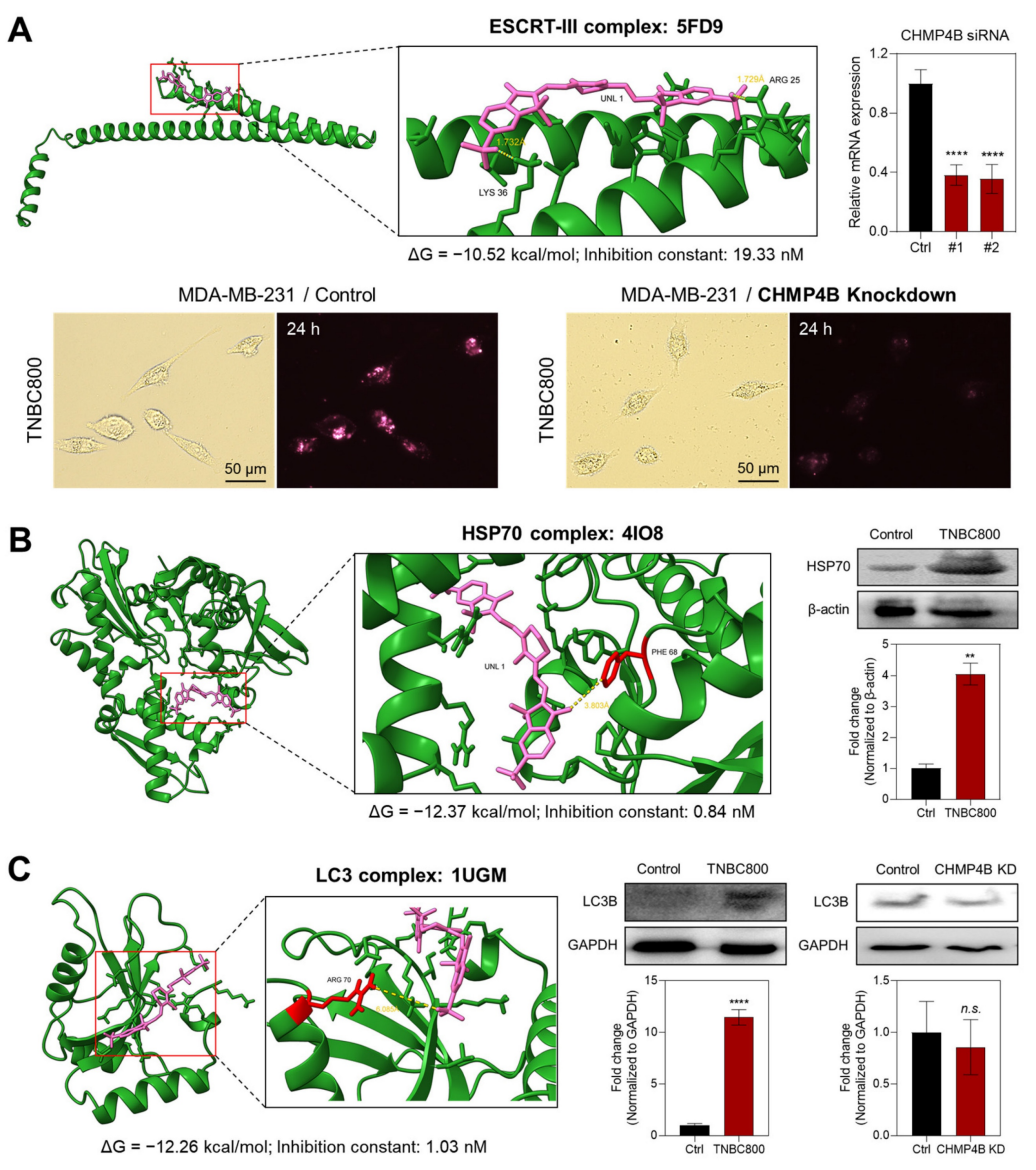
Molecular mechanism of TNBC800 in MDA-MB-231 cells and tumors. A) *In silico* molecular docking and prediction of a binding interaction between TNBC800 and the ESCRT-III complex (PDB ID: 5FD9). Cellular uptake of TNBC800 in MDA-MB-231 cells was evaluated before and after CHMP4B knockdown. The CHMP4B-silenced cells were incubated with 20 μM TNBC800 for 24 h before imaging. Scale bars = 50 μm. Real-time quantitative PCR was performed to confirm the efficiency of CHMP4B knockdown in MDA-MB-231 cells following transient siRNA transfection. B) *In silico* molecular docking and prediction of a binding interaction between TNBC800 and the HSP70 complex (PDB ID: 4IO8). Western blot and quantitative analysis of HSP70 in tumor tissues harvested from mice at day 7 after treatment with TNBC800. β-actin was used as a loading control. C) *In silico* molecular docking and prediction of a binding interaction between TNBC800 and the LC3 complex (PDB ID: 1UGM). Western blot and quantitative analysis of LC3B in tumor tissues harvested from mice at day 7 after treatment with TNBC800. The MDA-MB-231 cells after CHMP4B knockdown were incubated with 20 μM of TNBC800 for 24 h. GAPDH was employed as the loading standard. Values are expressed as mean ± S.D. (^**^p < 0.01, ^****^p < 0.0001, *n* = 3).

After confirming the localization of TNBC800 to lysosomes, molecular docking simulations were conducted using AutoDockTools (v1.5.7) to predict the intracellular uptake mechanism of TNBC800. Since it is known that the endosomal sorting complex required for transport (ESCRT) machinery is involved in a wide range of membrane-associated processes, including endocytosis, exocytosis, and autophagy, malfunctions in the ESCRT system are implicated especially in lysosomal storage disorders, leading to the secondary impairment of autophagy 23. Based on the molecular docking simulations between TNBC800 and the ESCRT complexes, TNBC800 showed the best binding affinity (ΔG = -10.52 kcal/mol) and inhibition constant (K_i_ = 19.33 nM) with the ESCRT-III complex (PDB ID: 5FD9) through hydrogen bonding interactions with ARG 25 (1.729 Å) and LYS 36 (1.732 Å) residues, while ZW800-Cl displayed lower binding affinity (ΔG = -7.32 kcal/mol) and inhibition constant (K_i_ = 4.29 µM) under the same condition (Figures 3A and S5A). Thus, we confirmed the role of ESCRT pathway proteins, especially the ESCRT-III complex, in lysosomal targeting of TNBC800 after knockdown of the CHMP4B gene in MDA-MB-231 cells, because the CHMP4B is a core component of the ESCRT-III complex (Figure S6). Importantly, the NIR fluorescence intensities of TNBC800 were significantly diminished in the MDA-MB-231 cells after CHMP4B knockdown, while the strong fluorescence signals were observed in the typical MDA-MB-231 cells treated with TNBC800. This result is consistent with *in silico* binding affinity prediction and also suggests that the ESCRT pathway is involved in the internalization and intracellular trafficking of TNBC800. Moreover, the specific binding affinity between TNBC800 and CHMP4B was directly confirmed by the immunoprecipitation assay using fluorescence and western blot analysis. Interestingly, the immunoprecipitated CHMP4B proteins from the MDA-MB-231 cells revealed strong NIR emission intensities only in the TNBC800-treated group, whereas no NIR fluorescence signals were detected in other groups (Figure S7A). For the final step, the immunoprecipitated CHMP4B proteins were verified by western blotting (Figure S7B). Additionally, the binding specificity between TNBC800 and CHMP4B was reconfirmed using the MDA-MB-231 xenografted tumors. As expected, enhanced NIR fluorescence signals of TNBC800 were clearly detected in the immunoprecipitated CHMP4B proteins, demonstrating the CHMP4B-binding specificity of TNBC800 *in vivo* (Figure S7C).

Since previous studies have demonstrated that HSP70 influences autophagy via various interconnected mechanisms, such as chaperone-mediated autophagy 24, we conducted the molecular docking simulations between TNBC800 and the HSP70 protein. As shown in Figures 3B and S5B, TNBC800 exhibited the binding affinity (ΔG = -12.37 kcal/mol) and inhibition constant (K_i_ = 0.84 nM) with the HSP70 complex (PDB ID: 4IO8) through hydrogen bonding interactions with the PHE 68 (3.803 Å) residue, whereas ZW800-Cl showed lower binding affinity (ΔG = -10.05 kcal/mol) and inhibition constant (K_i_ = 42.68 nM) under the same condition. Additionally, we examined the expression of HSP70 proteins in tumors collected from mice treated with TNBC800 using western blotting. Remarkably, the expression level of HSP70 was also statistically elevated in the tumors injected with TNBC800, comparing to the control group, indicating suppression of the autophagic response. As the effect of HSP70 overexpression, HSP70 can translocate to plasma membrane or be released into the extracellular space, it may take on an additional stress-inducible function, including the ability to trigger antitumor immune responses 25. Furthermore, the specific binding affinity between TNBC800 and HSP70 was directly identified by the immunoprecipitation assay using fluorescence and western blotting. As expected, the immunoprecipitated HSP70 proteins from the MDA-MB-231 cells displayed strong NIR fluorescent labels only in the TNBC800-treated group, while no NIR fluorescence signals were detected in other groups (Figure S8A). For the final step, the immunoprecipitated HSP70 proteins were confirmed by western blotting (Figure S8B).

To investigate the expression levels of proteins involved in autophagy, we also performed the molecular docking simulations between TNBC800 and the LC3 protein, which is a key protein in the autophagy pathway that acts as a marker for autophagosomes. As shown in Figures 3C and S5C, TNBC800 displayed the binding affinity (ΔG = -12.26 kcal/mol) and inhibition constant (K_i_ = 1.03 nM) with the LC3 complex (PDB ID: 1UGM) through hydrogen bonding interactions with the ARG 70 (6.085 Å) residue, while ZW800-Cl exhibited lower binding affinity (ΔG = -6.08 kcal/mol) and inhibition constant (K_i_ = 35.07 µM) under the same condition. To verify the role of LC3B as the core autophagic machinery, we further confirmed the expression of LC3B proteins in tumors harvested from mice treated with TNBC800 using western blotting. Importantly, the level of LC3B expression was substantially increased in the TNBC800-treated tumors, demonstrating induction of excessive autophagy. Previously, it was demonstrated that raising the activation threshold of LC3B confers a pro-apoptotic role that consequently inhibits the breast tumor growth 26. Additionally, the expression level of LC3B in the MDA-MB-231 cells after CHMP4B knockdown followed by treatment of TNBC800 was reconfirmed using western blot analysis. As anticipated, the level of LC3B expression in the CHMP4B knockdown cells revealed no significant differences in both groups, suggesting that TNBC800 could induce the lethal autophagy, thereby contributing to apoptotic cell death.

### *In vivo* tumor accumulation and antitumor efficacy of TNBC800

**Figure 4:**
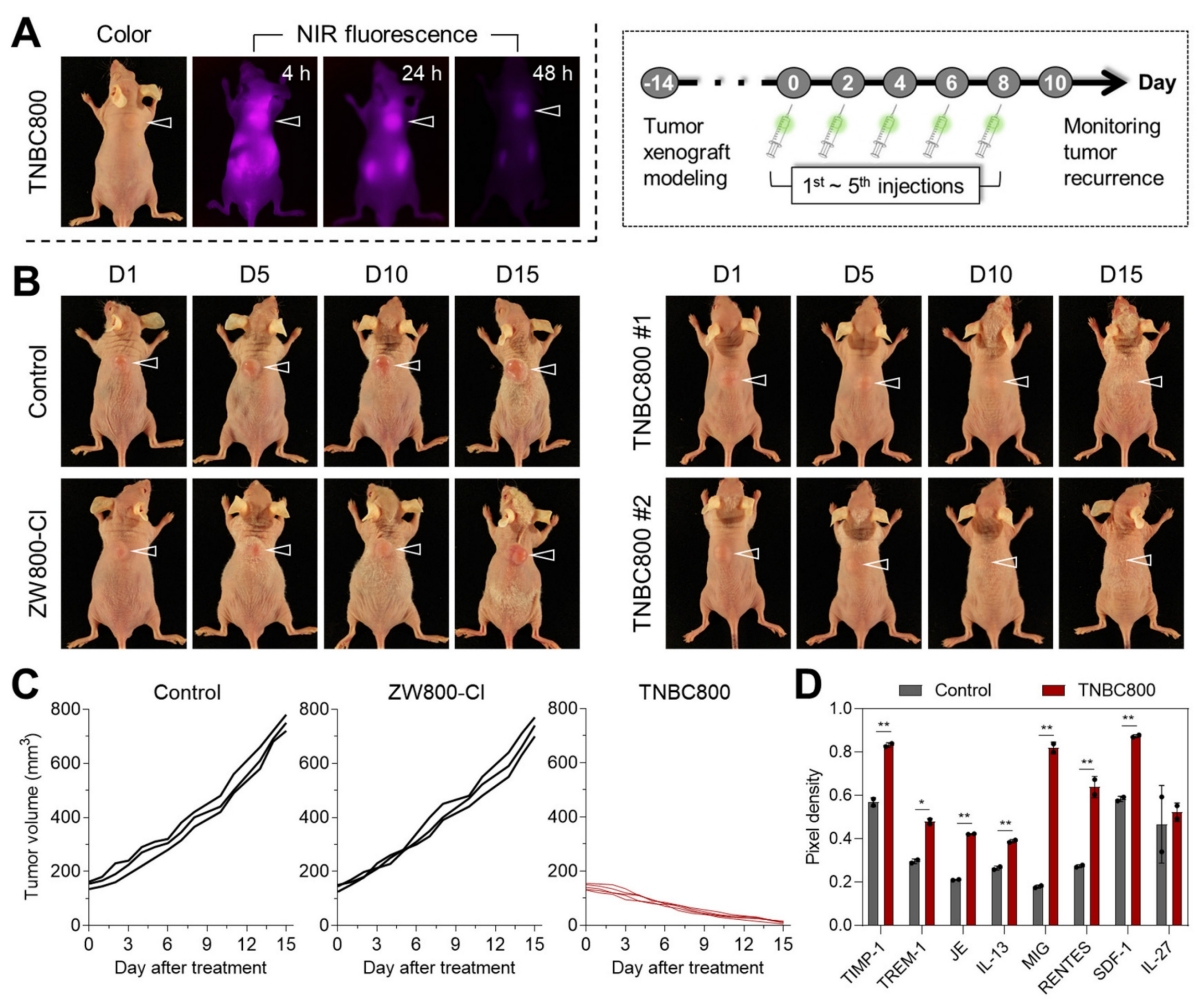
The tumor accumulation and *in vivo* performance of TNBC800. A) Serial NIR fluorescence imaging over 48 h following TNBC800 administration. MDA-MB-231 tumor-bearing mice received intravenous TNBC800 (1.0 mg kg^-1^, *n* = 3), with tumor location marked by arrowheads. B) Therapeutic response to TNBC800 in the MDA-MB-231 xenograft model. Mice were given intravenous TNBC800 (1.0 mg kg^-1^, *n* = 5) or ZW800-Cl (1.3 mg kg^-1^, *n* = 3) at 2-day intervals (5 total doses over 8 days). Tumor position indicated by arrowheads. C) Tumor progression curves tracked over 15 days for each treatment group. D) Multiplex cytokine profiling in mouse serum following TNBC800 therapy. Serum was collected on day 3 of treatment, and cytokines were measured in duplicate using a mouse cytokine array. Pixel intensities, quantified by ImageJ, reflect relative cytokine concentrations. Values are expressed as mean ± S.D. (^*^p < 0.05, ^**^p < 0.01, *n* = 3).

Following intravenous administration, the *in vivo* tumor-homing ability of TNBC800 was evaluated in MDA-MB-231 xenograft-bearing mice, as shown in Figures 4A and S9A. Real-time NIR fluorescence imaging was used to track tumor accumulation and signal retention of TNBC800 for up to 48 h after injection. As expected, TNBC800 showed strong tumor localization in mice. Also, the NIR fluorescent intensity at the tumor area was reduced over time during the 48 h observation period. To investigate its distribution and elimination profiles, the NIR fluorescence imaging was carried out at 24 and 48 h of injection, respectively, after collecting organs and tissues (Figure S9B,C). Most of the administered TNBC800 was cleared from the body within 48 h, suggesting a low risk of systemic toxicity. After establishing its tumor retention behavior, TNBC800 was then administered to MDA-MB-231 xenograft mice every 2 days for a total of five doses over 10 days. Tumor growth was monitored for 15 days to assess antitumor efficacy. Compared with the ZW800-Cl group, TNBC800 produced a pronounced suppression of tumor expansion, whereas ZW800-Cl showed little to no inhibitory activity and remained comparable to the control group (Figure 4B,C). The antitumor effect of TNBC800 was further confirmed in a BALB/c 4T1 allograft model under the same dosing schedule (five administrations over 10 days), with tumor progression monitored for 15 days. In this model as well, TNBC800 effectively restrained tumor growth comparing to the control group (Figure S10A). This indicates that TNBC800 may trigger lethal autophagy and thereby exert therapeutic activity against TNBC.

To assess *in vivo* safety, major organs, including the heart, lung, liver, spleen, and kidney, were collected on day 15 for hematoxylin & eosin (H&E) staining. Distinct tissue damage or histological abnormalities were not detected in mice injected with TNBC800 (Figure S11A). In addition, serum chemistry parameters were analyzed from blood samples collected on day 15 (Figure S11B). No significant differences were observed between the TNBC800-treated and control groups, indicating that TNBC800 has favorable biosafety and may be suitable for future translational development. To clarify whether inflammatory signaling contributed to the antitumor response, serum samples were collected on day 7 of treatment and analyzed for cytokine expression (Figure S12). Since pro-inflammatory cytokines are key mediators of immune regulation, the cytokine array showed elevated levels of TREM-1, MIG (CXCL9), and RANTES (CCL5), implying activation of immune signaling by TNBC800 (Figure 4D).

**Figure 5:**
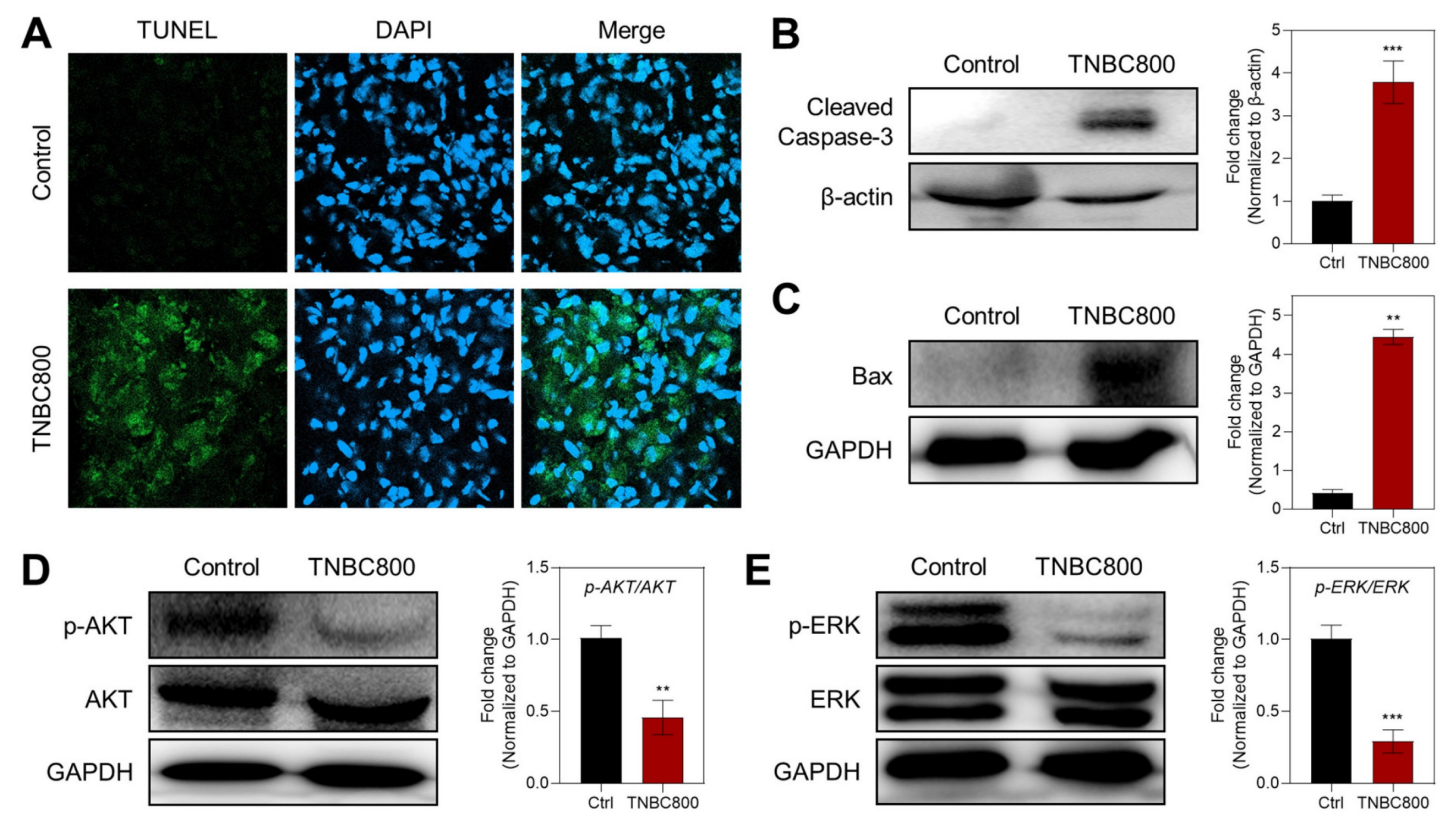
TNBC800-mediated antitumor effects. A) TUNEL staining to visualize apoptotic cells in tumor sections collected from mice on day 7 post-TNBC800 treatment. DAPI counterstaining highlights cell nuclei. Western blot analysis and densitometric quantification of key apoptosis regulators in xenograft tumors: B) c-Caspase-3, C) Bax, D) p-AKT, and E) p-ERK. GAPDH or β-actin served as loading controls. Values are represented as mean ± S.D. (^**^p < 0.01, ^***^p < 0.001, *n* = 3).

The antitumor effect of TNBC800 was also supported by TUNEL staining of tumor samples collected on day 7, which is commonly used to detect apoptotic cells (Figure 5A). Strong green fluorescence was evident in the TNBC800-treated tumors, whereas little signal was detected in the control group, indicating that TNBC800 effectively inhibited tumor proliferation while promoting apoptosis. Consistent with this, western blot analysis of tumor lysates from MDA-MB-231 xenografts on day 7 showed elevated expression of cleaved caspase-3 and Bax in the TNBC800 group relative to controls (Figure 5B,C). To further examine the mechanism underlying TNBC800-induced cell death, the phosphorylation status of AKT and ERK was analyzed by western blotting. Both p-AKT and p-ERK levels were markedly reduced in TNBC800-treated MDA-MB-231 tumors (Figure 5D,E). Because the AKT/mTOR and MAPK pathways are important not only for proliferation and differentiation but also as negative regulators of autophagy 27, these findings indicate that TNBC800 induces apoptotic death specifically in MDA-MB-231 xenografted tumors.

### Antitumor immunity induced by TNBC800

**Figure 6:**
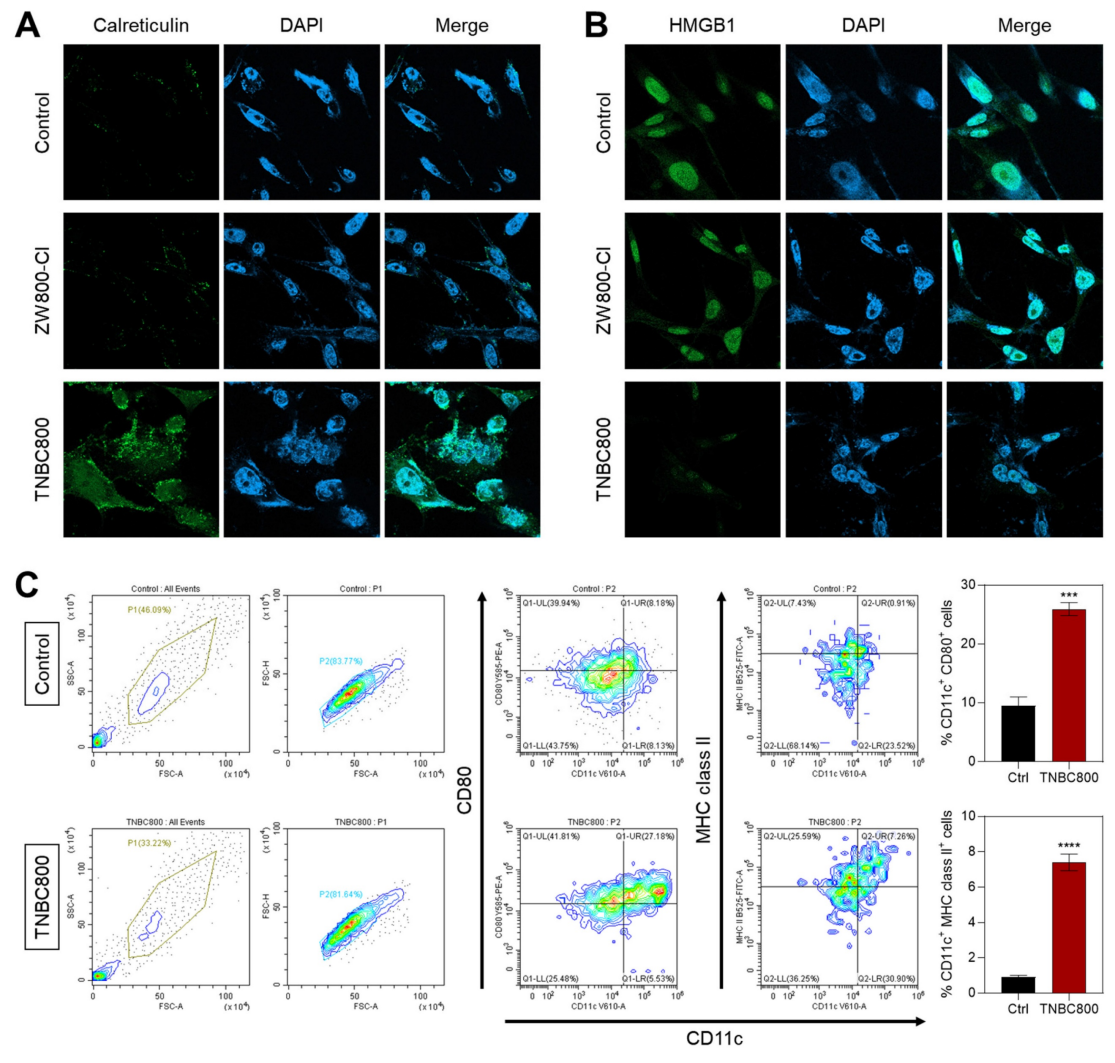
Quantitative assessment of key DAMPs following TNBC800 or ZW800-Cl treatment in xenografts. Confocal micrographs illustrating immunofluorescence detection of A) calreticulin and B) HMGB1 in tumor sections. Nuclei were counterstained with DAPI (blue). All fluorescence images were captured with uniform exposure settings and normalization. C) Flow cytometric evaluation and percentage of mature dendritic cells (CD80^+^ MHC class II^+^) in BMDCs following TNBC800 exposure. Values are expressed as mean ± S.D. (^***^p < 0.001, ^****^p < 0.0001, *n* = 3).

Given the notable antitumor activity of TNBC800 confirmed in MDA-MB-231 xenograft mice, we further explored whether this hydrophilic, anionic small-molecule dye could provoke immune responses that enhance tumor-specific immunity. During ICD, dying tumor cells release various damage-associated molecular patterns (DAMPs) that promote the recruitment and activation of dendritic cells. Among these, calreticulin relocates from the endoplasmic reticulum to the plasma membrane in response to ROS-mediated endoplasmic reticulum stress, acting as a pro-phagocytic signal recognized by immune cells 28. Immunofluorescence and confocal imaging revealed a pronounced increase in surface calreticulin levels in TNBC800-treated MDA-MB-231 xenograft tumors compared to those receiving ZW800-Cl (Figure 6A). In addition, release of high-mobility group box 1 (HMGB1) was confirmed by immunofluorescent staining in tumors after treatment with either ZW800-Cl or TNBC800 (Figure 6B). Tumors exposed to TNBC800 displayed noticeably reduced intracellular HMGB1 fluorescence compared to the control group, demonstrating effective release of HMGB1 into the extracellular space, leading to a strong ICD response in the MDA-MB-231 xenografts.

To further assess the immune responses triggered by TNBC800, we evaluated dendritic cell (DC) activity in the presence of TNBC800. DCs are pivotal mediators of adaptive immunity, and ICD-induced signals promote their recruitment and maturation, ultimately leading to T-cell activation and antitumor cytotoxicity. Mature DCs are characterized by high levels of surface MHC II or costimulatory molecules (CD86 and CD80) 29. Flow cytometric analysis of bone marrow-derived dendritic cells (BMDCs) after 24 h of TNBC800 exposure revealed significant upregulation of maturation markers CD80^+^ and MHC class II^+^ by 2.8-fold and 7.5-fold, respectively, compared with untreated controls (Figure 6C).

**Figure 7:**
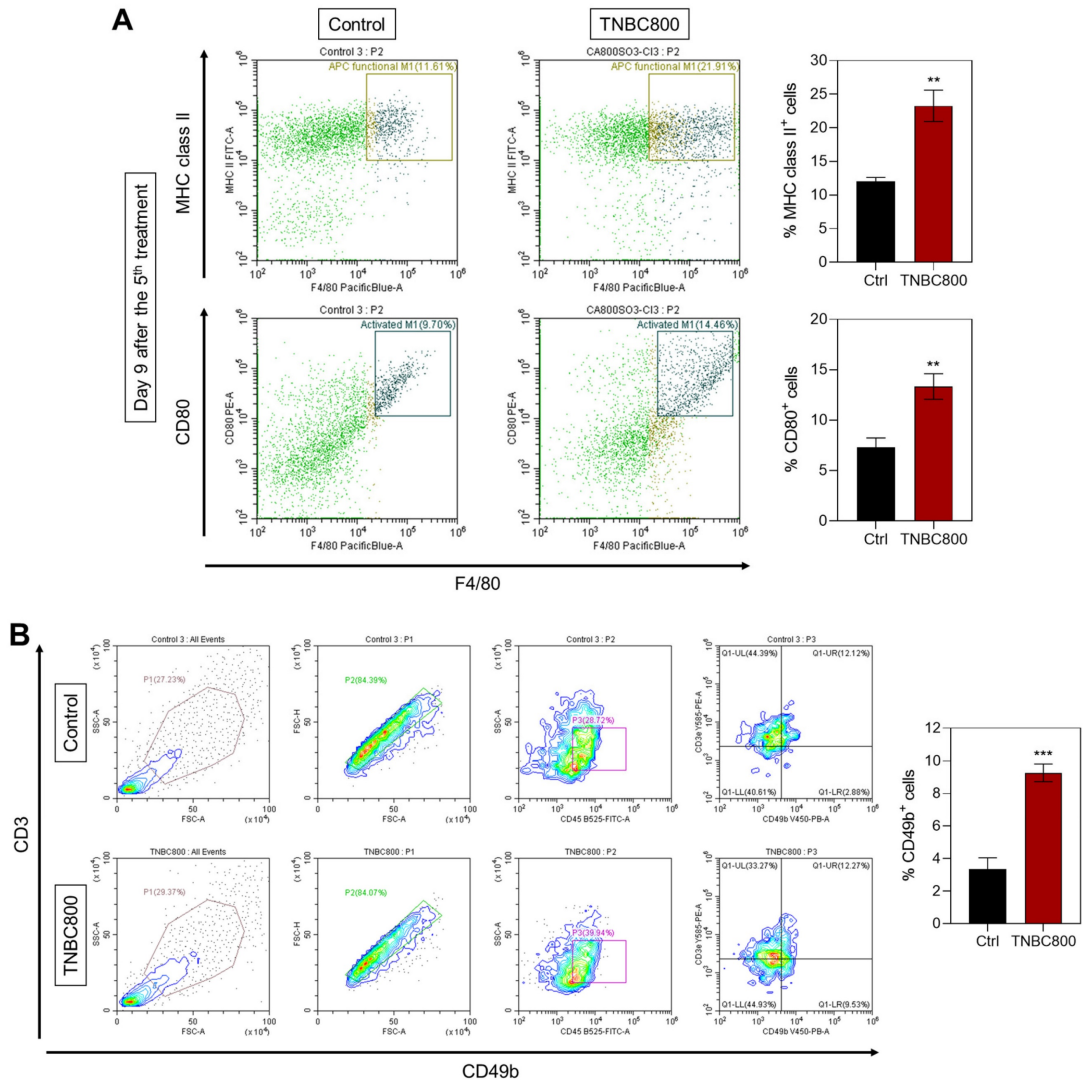
TNBC800-induced antitumor immune responses. A) Flow cytometric analysis and percentage of M1-like macrophages in spleen tissue collected on day 9 following the fifth TNBC800 dose. Values are represented as mean ± S.D. (^**^p < 0.01, *n* = 3). B) Flow cytometric evaluation and proportion of NK cells (CD49b^+^) in tumor tissue obtained on day 3 after the second TNBC800 administration. Values are expressed as mean ± S.D. (^***^p < 0.001, *n* = 3).

Since M1-polarized macrophages similarly express MHC class II and CD80, both related to antigen presentation and T-cell activation, we next confirmed expression of M1 markers in spleen tissues on day 9 after TNBC800 administration. A marked increase in the expression of MHC class II and CD80 was detected relative to control samples (Figure 7A). Furthermore, immunofluorescence staining of tumors collected three days post-treatment demonstrated elevated expression of NK cell marker CD49b in both MDA-MB-231 and 4T1 tumor models (Figures 7B and S10B). This pronounced NK cell infiltration highlights an active immune engagement following TNBC800 treatment. Collectively, these findings suggest that TNBC800-induced autophagic and immunogenic stress enhances dendritic cell maturation, boosts M1 macrophage presence, and fosters immune cell infiltration, together contributing to remodeling of the immunosuppressive tumor microenvironment. Therefore, TNBC800 promotes antitumor immunity by initiating ICD and elevating pro-inflammatory cytokine responses, offering a promising and biocompatible strategy for cancer immunotherapy.

## Conclusion

In this study, we developed a novel heptamethine cyanine dye, TNBC800, designed for an efficient and straightforward therapeutic approach against TNBC. Leveraging the principle of “structure-inherent cancer targeting,” the hydrophilic anionic dye TNBC800 demonstrated selective accumulation in tumors, enabling NIR fluorescence imaging alongside targeted antitumor effects in both MDA-MB-231 and 4T1 mouse models. TNBC800 triggered pronounced autophagic stress and elevated intracellular ROS in MDA-MB-231 cells. This intracellular perturbation subsequently promoted the release of DAMPs into the extracellular milieu, which in turn activated DCs and enhanced antitumor immune responses. As a result, we observed a notable enrichment of proinflammatory M1 macrophages in spleen and NK cells within the tumor microenvironment. Overall, this work highlights a practical strategy for designing multifunctional small-molecule theranostic agents and provides mechanistic insight into how autophagy-mediated cellular stress can initiate ICD in both MDA-MB-231 cells and xenografted tumors.

## Materials and Methods

### Synthesis of TNBC800

All materials and instruments used in this study were listed in Tables S1 and S2. As illustrated in Figure 1A, the procedures for synthesizing TNBC800 and ZW800-Cl followed previously reported methods 15. The preparation of the hydrophilic heptamethine cyanine dye TNBC800 was carried out according to the following protocol:

2-[(E)-2-[(3E)-2-chloro-3-{2-[(2E)-1,3,3-trimethyl-5-sulfonato-2,3-dihydro-1H-indol-2-ylidene]ethylidene}cyclohex-1-en-1-yl]ethenyl]-1,3,3-trimethyl-3H-indol-1-ium-5-sulfonate iodide (**TNBC800**). Phenylhydrazine-4-sulfonic acid **1** (3 g, 0.016 mol) and isopropyl methyl ketone **2** (2.3 mL, 0.021 mol) were mixed in acetic acid (30 mL) and heated up to 120 °C for 18 h. After completion, the reaction mixture was allowed to cool, and the precipitate was obtained by filtration. The crude product was further precipitated from ethyl acetate, affording a pink solid (3.2 g, 79%). Subsequently, 2,3,3-trimethyl-3H-indole-5-sulfonate **3** (0.1 g, 0.42 mmol) and iodomethane **4** (0.07 g, 0.49 mmol) were mixed in toluene (4 mL) and heated up to 70 °C under nitrogen for 48 h. Upon cooling to ambient temperature, the solvent was removed by decantation, and the residue was recrystallized from acetonitrile to yield a solid used directly in the next reaction (0.07 g, 69%). Finally, the compound **5** (0.18 g, 0.7 mmol), N-{[(3E)-3-(anilinomethylene)-2-chlorocyclohex-1-en-1-yl]methylene}benzen-aminium chloride **6** (0.1 g, 0.3 mmol), and acetic acid sodium salt (0.08 g, 0.98 mmol) were mixed in ethyl alcohol (15 mL) and refluxed for 8 h. After cooling, the reaction solution was isolated by filtration, washed with ethyl acetate, and dried to afford a green solid identified as TNBC800 (0.15 g, 83%). Exact mass (ESI) m/z [M]^+^ calculated for [C_32_H_34_ClN_2_O_6_S_2_]^+^ 641.1552, detected [M]^+^ 641.1559.

### Optical analysis and physicochemical properties

Optical property measurement of TNBC800 and ZW800-Cl was carried out after dissolving in PBS. The absorbance of TNBC800 and ZW800-Cl was measured respectively and used to calculate molar extinction coefficients according to the Beer-Lambert law. NIR fluorescence spectra for TNBC800 and ZW800-Cl were measured respectively and used to determine fluorescence quantum yields (*Φ*) relative to the clinically available indocyanine green 30. Computational estimations of the hydrophobicity (log*D*) and TPSA were carried out using the Chemaxon Marvin software.

### Cytotoxicity assay

MDA-MB-231, MCF-7, 4T1, and NIH/3T3 cell lines were used in this study. MDA-MB-231 cells were authenticated using short tandem repeat (STR) profiling (Figure S13). Cells were maintained under standard two-dimensional (2D) culture conditions in RPMI 1640 medium supplemented with 10% heat-inactivated fetal bovine serum (FBS) and antibiotics. Multiple cell batches were employed to ensure reproducibility. Cultures were kept in a humidified incubator at 37 °C with 5% CO_2_. When cells reached roughly 80% confluence, viability and proliferation were determined via MTT assay. For this, cells were cultured on 96-well plates with 5 × 10^3^ cells per well. After that, TNBC800 was treated at 2-50 μM concentrations for 24 h. After incubation, the 10 μL of MTT solution was added into each well. Plates were incubated at 37 °C in a 5% CO_2_ humidified atmosphere for 4 h. Formazan crystals were then solubilized using dimethyl sulfoxide, and absorbance was recorded at 570 nm with a microplate reader.

### Live-cell fluorescence imaging

MDA-MB-231 cells were incubated with 20 μM TNBC800 for 24 h at 37 °C in a humidified atmosphere containing 5% CO_2_. Following treatment, the Golgi apparatus, mitochondria, and lysosomes were labeled using commercially available tracking dyes, respectively, for 5-30 min in accordance with the supplier's protocols. After staining, the stained cells were rinsed with DPBS and visualized. The images were captured using consistent exposure settings and normalized for comparison.

### Fluorometric intracellular ROS assay

ZW800-Cl and TNBC800 (20 μM) were treated in the MDA-MB-231 cells for 24 h. After treatment, the cells were rinsed using PBS and incubated with 100 μM of DCF-DA for a half an hour. The DCF-DA probe, initially nonfluorescent, penetrates the cell membrane and is converted by ROS into the green fluorescent DCF. After washing with PBS, the MDA-MB-231 cells were examined using a fluorescence microscope.

### Small interfering RNA transfection

To suppress CHMP4B expression, MDA-MB-231 cells were subjected to small interfering RNA (siRNA) transfection. Cells were plated in 6-well culture dishes and transfected with 2.5 μg of the indicated plasmid using Lipofectamine^TM^ 3000 Reagent. Six hours after transfection, the medium containing the transfection reagents was replaced with RPMI 1640 supplemented with 10% FBS, and cells were cultured for an additional 48 h. The siRNA sequences used were: siCHMP4B #1: 5′-CGG AAG AGA UGU UAA GCA A-3′; siCHMP4B #2: 5′-CGA UAA AGU UGA UGA GUU A-3′. Transfection efficiency was evaluated using both fluorescence microscopy and quantitative PCR analysis.

### Quantitative PCR

TRIzol reagent was used to extract total RNA according to the supplier's protocol. Subsequently, total RNA (1 μg) was converted into complementary DNA utilizing RevertAid Reverse Transcriptase and random hexamer primers. The synthesized complementary DNA served as a template for quantitative PCR (qPCR). Amplification was conducted using nTaq (Mg2+Plus) DNA polymerase with gene-specific primers. The primer sequences were as follows: CHMP4B (forward: 5′-CCC TCT ACC AAA TGT TCC CTC-3′; reverse: 5′-CCC AGT TCT CCA ATT CCT TCA-3′), GAPDH (forward: 5′-AAT CCC ATC ACC ATC TTC CAG-3′; reverse: 5′-TTC ACA CCC ATG ACG AAC AT-3′). A standard curve was established using serial dilutions of complementary DNA to determine the relative expression levels of each target gene. Expression data were normalized to GAPDH as the internal control. All qPCR reactions were performed in triplicate and independently repeated three times, yielding consistent outcomes.

### *In silico* molecular docking simulation

The three-dimensional (3D) crystal structures of the proteins were obtained from the RCSB Protein Data Bank (PDB IDs: 5FD9, 4IO8, and 1UGM). The initial structures were processed using UCSF ChimeraX (v1.9), which can remove unnecessary residues, chains, and water molecules. The modified proteins were imported into ADT. Kollman charges were computed, non-polar hydrogens were merged, and AutoDock4 atoms were assigned. The ligand, TNBC800, was initially drawn as a 2D structure using ChemDraw Ultra 8.0. Optimization and 3D conversion of TNBC800 were processed using Avogadro v1.2.0. The ligand was saved as a MOL2 file and subsequently converted to the PDBQT format using AutoDockTools (v1.5.7). For each docking simulation, the grid box dimensions were set to 126 points in each of the x, y, and z dimensions, with a spacing of 0.547 Å between the grid points. The docking parameters were performed using the Lamarckian Genetic Algorithm. Default docking settings were used for simulation. Visualization of the protein-ligand interactions, including hydrogen bonds and amino acids, was performed using UCSF ChimeraX (v1.9).

### Immunoprecipitation assay

To confirm specific molecular interactions between TNBC800 and CHMP4B or HSP70, MDA-MB-231 cells or xenografted tumors were collected and lysed with NP-40, followed by addition of 200 µM of TNBC800, anti-CHMP4B antibody, anti-HSP70 antibody or Normal Rabbit IgG antibody to the lysate for 12 h at 4 °C. Then, the lysates were stored with protein A/G magnetic beads for 1 h. After washing the magnetic beads using TBS-T (0.05% of Tween-20), the lysates were treated with fluorophore-conjugated secondary antibodies and then imaged using a fluorescence microscope. Subsequently, a low pH elution buffer and a neutralization buffer were added to separate the magnetic beads. Finally, the immunoprecipitated proteins were detected by western blot.

### Xenograft and syngeneic mouse models

All animal procedures were performed following the protocols authorized by the Chonnam National University Institutional Animal Care and Use Committee (CNU IACUC-H-2024-75). Mice were used to generate syngeneic tumor and human xenograft models, respectively. MDA-MB-231 or 4T1 cells cultured *in vitro* were suspended in PBS and injected subcutaneously into the right flank of each nude or BALB/c mouse at a dose of 2 × 10^6^ cells per animal. When subcutaneous tumors reached an average size of about 1 cm in diameter, mice received intravenous injections of either TNBC800 or ZW800-Cl. At predetermined time points, tumor-bearing mice were anesthetized and subjected to real-time, whole-body NIR fluorescence imaging. Groups of three to five mice per treatment condition were monitored for up to 48 h of injection to assess the tumor targeting efficiency of TNBC800. Fluorescence intensity was normalized uniformly across all imaging datasets. Antitumor efficacy was further evaluated by measuring tumor growth over a 15-day period, and tumor volume (V) was determined by the equation: V = 0.5 × longest diameter × (shortest diameter)^2^.

### Cytokine array

MDA-MB-231 xenograft-bearing mice were administered either PBS or TNBC800, and serum cytokine levels were analyzed using the Mouse Cytokine Array Kit following the supplier's protocol. In brief, total serum proteins were isolated and applied to array membranes pre-equilibrated with blocking buffer for 1 h. The membranes were then incubated with serum samples overnight at 4 °C, followed by washing and subsequent incubation with streptavidin-HRP conjugate for 2 h. Chemiluminescent signals were detected by a LuminoGraph III Lite imaging system. Spot intensities were analyzed by ImageJ software.

### TUNEL assay

Tumor samples from both control and TNBC800-treated mice were collected for measuring TUNEL staining. The tissues were fixed using ice cold 4% paraformaldehyde and subsequently sectioned with a cryostat. The sections were rinsed with PBS, treated with TUNEL reagent, and counterstained with DAPI for 30 min. Fluorescent images were then acquired using a Nikon fluorescence microscope.

### Western blotting

Whole-cell lysates and tumor extracts were obtained by disrupting the samples using RIPA buffer followed by homogenization. After centrifugation, the clarified supernatants were collected and mixed with Laemmli sample buffer, then heated at 95 °C for 5 min in a water bath to ensure complete protein denaturation. Equal amounts of protein (40 μg per lane) were loaded onto 8-15% SDS-polyacrylamide gels and subjected to polyacrylamide gel electrophoresis. The separated polypeptides were electroblotted to PVDF membranes pre-wetted with methanol. After that, 5% bovine serum albumin (BSA) was applied to the membranes at ambient temperature for 2 h, followed by incubation with primary antibodies (1:1000 dilution) for 12 h at 4 °C. The following primary antibodies were used: c-Caspase-3, HSP70, LC3B, Bax, p-AKT, AKT, p-ERK, ERK, GAPDH, and β-actin. After washing, membranes were incubated for 2 h at room temperature with HRP-conjugated secondary antibodies. Protein bands were visualized using an enhanced chemiluminescent detection reagent and imaged using the LuminoGraph III Lite system. Band intensities were quantified with ImageJ, and protein levels were normalized against GAPDH and β-actin.

### Immunofluorescence staining and confocal imaging

Tumors derived from MDA-MB-231 xenografts were collected from mice following TNBC800 treatment. The tumor tissue was fixed using ice cold 4% paraformaldehyde and subsequently sectioned with a cryostat. The cryosections were washed with PBS and permeabilized with 0.1% Triton X-100 for 5 min. To minimize nonspecific antibody binding, sections were incubated in 5% BSA for 1 h at room temperature. After PBS washing, samples were treated with primary antibodies and incubated for 12 h at 4 °C. The next day, sections were washed again with PBS and incubated with fluorescence-conjugated secondary antibodies, followed by counterstaining with DAPI for 5 min. Fluorescence images were obtained through a confocal microscope with a 63× objective lens. The primary antibodies used were anti-HMGB1 and anti-calreticulin.

### Flow cytometry analysis of BMDC maturation

BMDC was generated from BALB/c mice using a standard differentiation protocol. The obtained cells were resuspended in 10 mL of RPMI 1640 supplemented with 20 ng/mL GM-CSF and cultured at 37 °C in a humidified atmosphere containing 5% CO_2_. On day 3, 20 ng/mL GM-CSF in 10 mL of fresh culture medium was added, and the cultures were maintained for 3 more days. BMDCs were then collected from the non-adherent/loosely attached cell fraction. The harvested cells were incubated with 20 μM TNBC800 for 24 h, and maturation of BMDCs was finally assessed by a CytoFLEX instrument.

### Flow cytometry analysis of immune cell infiltration

After 9 days of PBS or TNBC800 treatment, MDA-MB-231 xenograft-bearing mice were euthanized, and the spleens and tumors were excised. Each tissue sample was minced into small fragments and mechanically dissociated on ice to prepare a tissue suspension. The suspension was then passed through 70 μm cell strainers to obtain single-cell suspensions, after which red blood cells were removed using lysis buffer and the cells were washed using PBS. The resulting cells were treated with fluorophore-conjugated antibodies following the supplier's protocol. F4/80, MHC class II, CD80, and CD49b were antibodies used (1:200 dilution). The labeled cells were identified by flow cytometry using a CytoFLEX instrument.

### *In vivo* evaluation of safety

To examine the systemic toxicity of TNBC800, major organs including the heart, liver, lungs, spleen, and kidneys were collected from each experimental group on day 15. The harvested tissues were fixed, prepared for H&E staining, and examined under a light microscope. In addition, blood samples were obtained on day 15 after TNBC800 administration. The samples were centrifuged at 4500 rpm for 10 min at 4 °C, and the separated serum was stored at -80 °C until biochemical analysis.

### Statistical analysis

Statistical evaluation was performed using one-way ANOVA followed by Tukey's multiple-comparison test. A *p* value below 0.05 was considered to be statistically significant. Data are reported as mean ± standard deviation (S.D.).

## Supplementary Material

Supplementary figures and tables.

## Figures and Tables

**Scheme 1:**
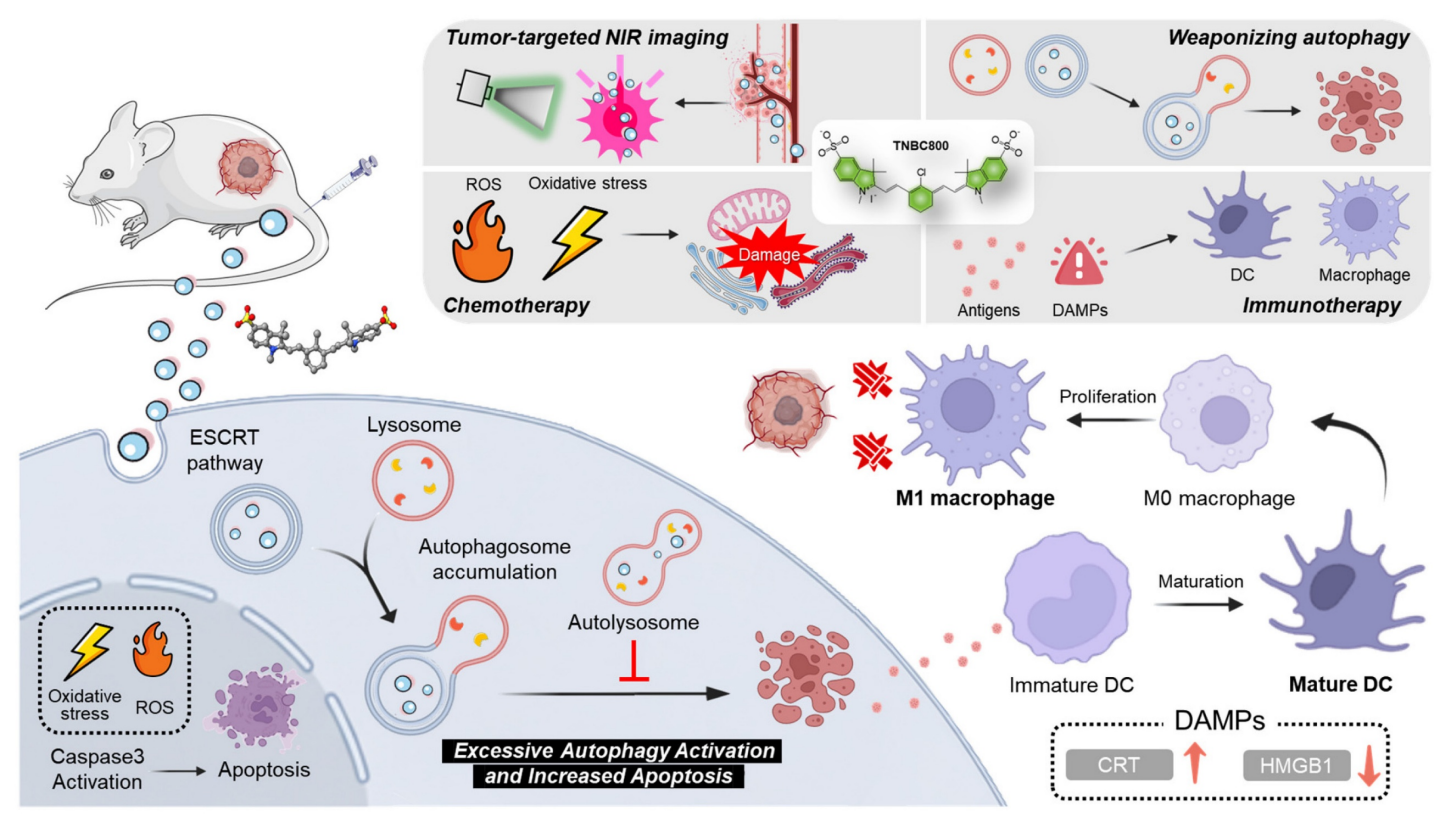
The molecular action mechanism of TNBC800. The tumor-targeted TNBC800 is localized in lysosomes through the ESCRT pathway to induce excessive activation of autophagy, thereby resulting in autophagosome accumulation leading to apoptosis. TNBC800 also induces the increase of intracellular ROS, triggering the initiation of ICD for the effective treatment of TNBC.

## Data Availability

The data that support the findings of this study are available from the corresponding author upon reasonable request.
