# Supplementary material for: A tumor-targeted heptamethine cyanine dye suppresses triple-negative breast cancer by induction of lethal autophagy
Source: Theranostics. 2026 Apr 23;16(11):6366–79. doi: 10.7150/thno.130353 (PMC13142667; doi:10.7150/thno.130353)
Supplement: Supplementary file 1 — Supplementary figures and tables. [file thnov16p6366s1.pdf]

A tumor-targeted heptamethine cyanine dye suppresses triple-negative breast cancer by induction of lethal autophagy

Sang-Hyo Kim[1,2,†], Yoonbin Park[1,2,†], Hwa-yeong Jin[1,2], Taewon Lee[3], Sungsu Lee[4,*], Moon Suk Kim[5,*], Hoon Hyun[1,2,*]

[1] Department of Biomedical Sciences, Chonnam National University Medical School, Hwasun 58128, South Korea
[2] BioMedical Sciences Graduate Program (BMSGP), Chonnam National University, Hwasun 58128, South Korea
[3] Division of Applied Mathematical Sciences, College of Science and Technology, Korea University, Sejong 30019, South Korea
[4] Department of Otolaryngology-Head and Neck Surgery, Chonnam National University Medical School, Gwangju 61469, South Korea
[5] Department of Molecular Science and Technology, Ajou University, Suwon 16499, South Korea

[†] Sang-Hyo Kim and Yoonbin Park contributed equally to this work.

[*] Corresponding authors: Sungsu Lee, E-mail: minsunglss@jnu.ac.kr; Moon Suk Kim, E-mail: moonskim@ajou.ac.kr; Hoon Hyun, E-mail: hhyun@jnu.ac.kr

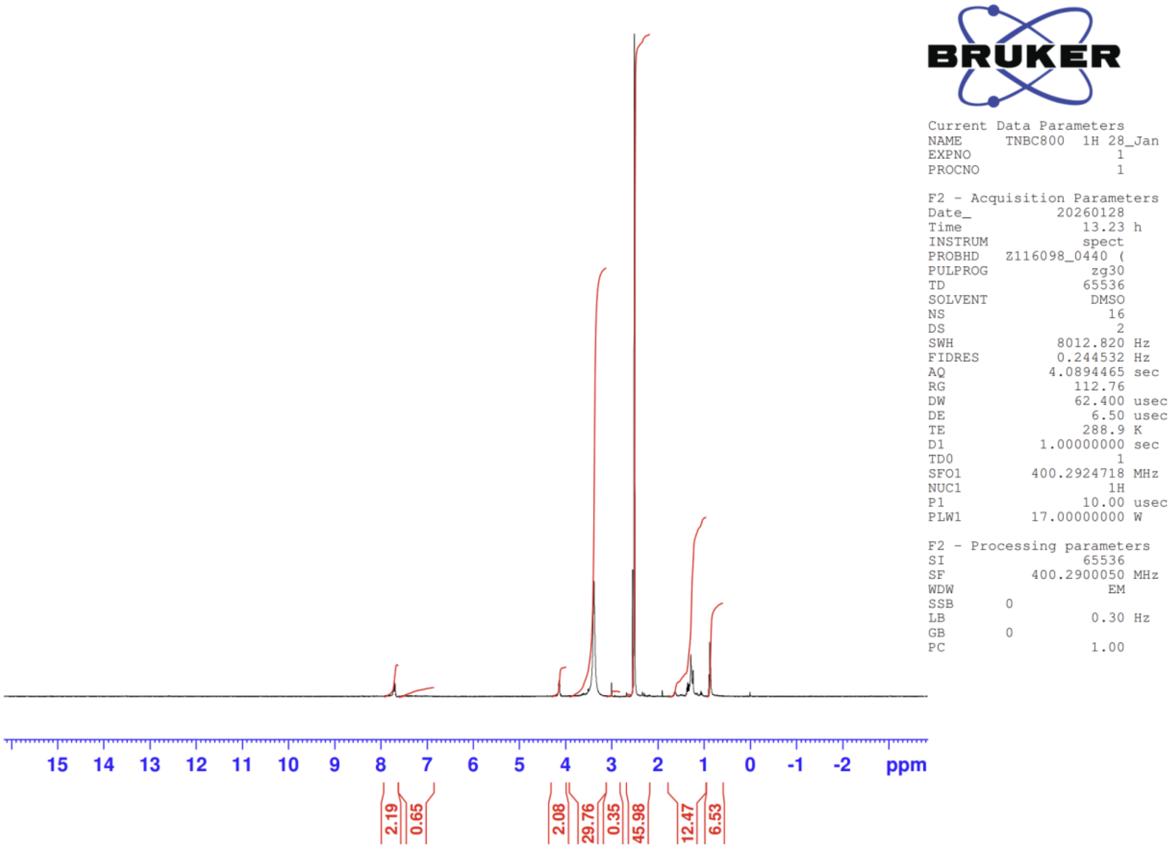

Figure S1. ^{1}H NMR spectrum of TNBC800.

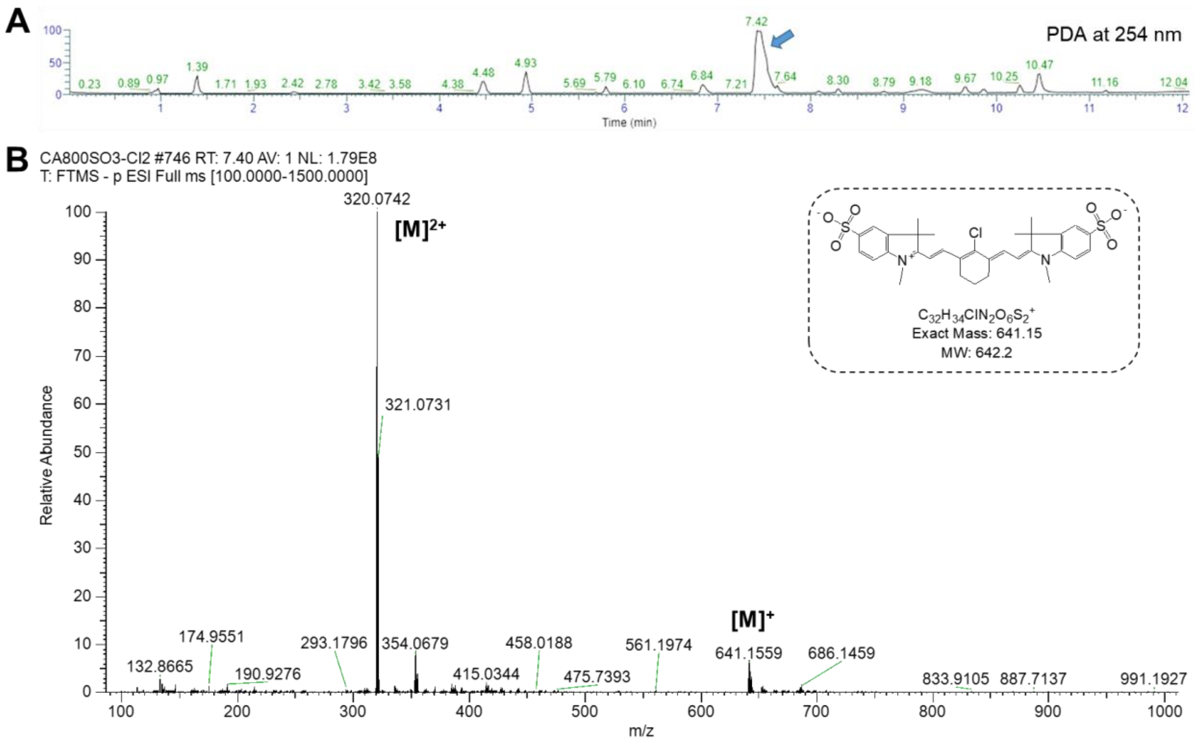

Figure S2. A) HPLC trace and B) mass spectrum of TNBC800.

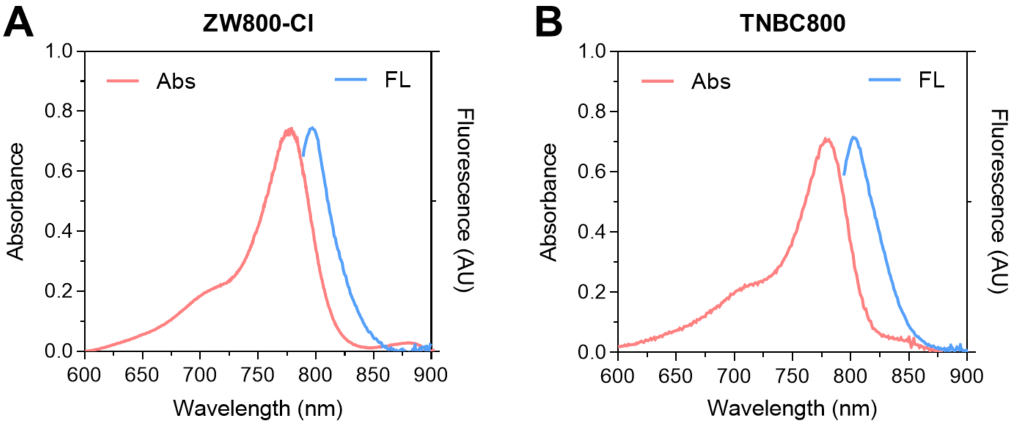

Figure S3. Absorption and fluorescence emission spectra of A) ZW800-Cl and B) TNBC800 measured in PBS at pH 7.4.

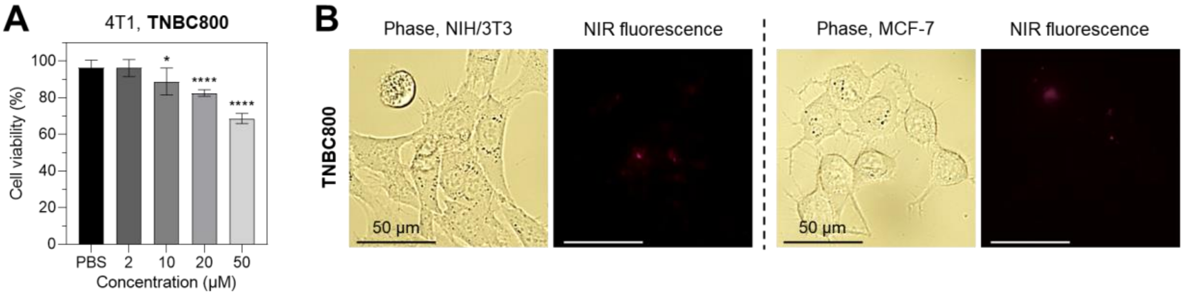

Figure S4. A) Cell viability assay of TNBC800 in 4T1 breast cancer cells. Percentage cytotoxicity is determined after 24 h of treatment with various concentrations of TNBC800. Data are expressed as mean ± S.D. ($^{*}p < 0.05$, $^{****}p < 0.0001$, $n = 6$). B) Live cell binding of TNBC800 in NIH/3T3 and MCF-7 cells. Phase contrast and NIR fluorescence images are obtained after 24 h of incubation with 20 µM of TNBC800, respectively. Images are representative of $n = 3$ independent experiments. All fluorescence images had identical exposure times and normalization. Scale bars = 50 µm.

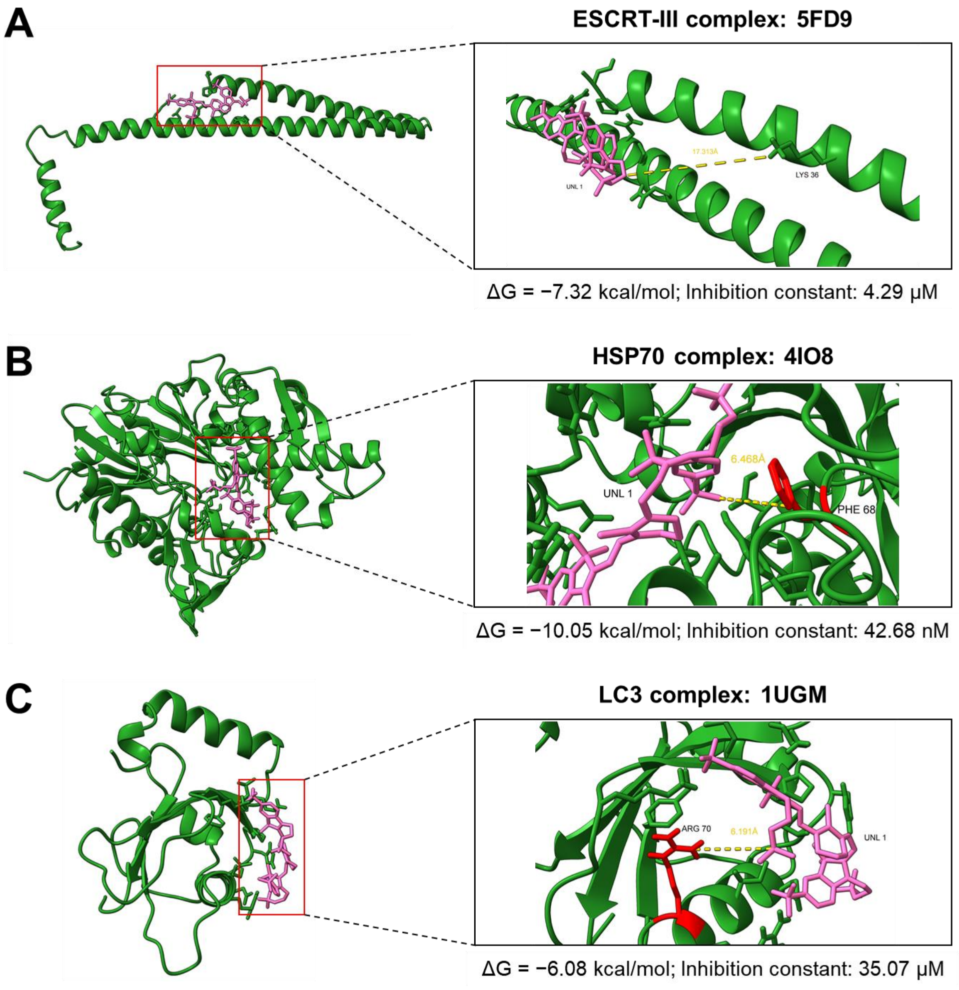

Figure S5. *In silico* molecular docking and prediction of binding interactions between ZW800-Cl and A) the ESCRT-III complex (PDB ID: 5FD9), B) the HSP70 complex (PDB ID: 4IO8), and C) the LC3 complex (PDB ID: 1UGM).

Name	Sequence (5’ ⟶ 3’)
CHMP4B_FWD_hg19	CCCTCTACCAAATGTTCCCTC
CHMP4B_REV_hg19	CCCAGTTCTCCAATTCCTTCA
CHMP4B_SP216 (#1)	Sense : CGGAAGAGAUGUUAAGCAA=UU<br>Anti-Sense : UUGCUUAACAUCUCUUCCG=UU
CHMP4B_SP496 (#2)	Sense : CGAUAAAGUUGAUGAGUUA=UU<br>Anti-Sense : UAACUCAUCAACUUUAUCG=UU

Figure S6. Sequences of oligonucleotides for the expression of CHMP4B. The synthetic oligonucleotides are designed to involve the targeted depletion of CHMP4B. For gene silencing, a small interfering RNA (siRNA) specific to CHMP4B was employed, alongside a non-targeting siRNA as a procedural negative control. The efficiency of siRNAs was validated by quantitative real-time PCR, normalizing target gene expression to GAPDH.

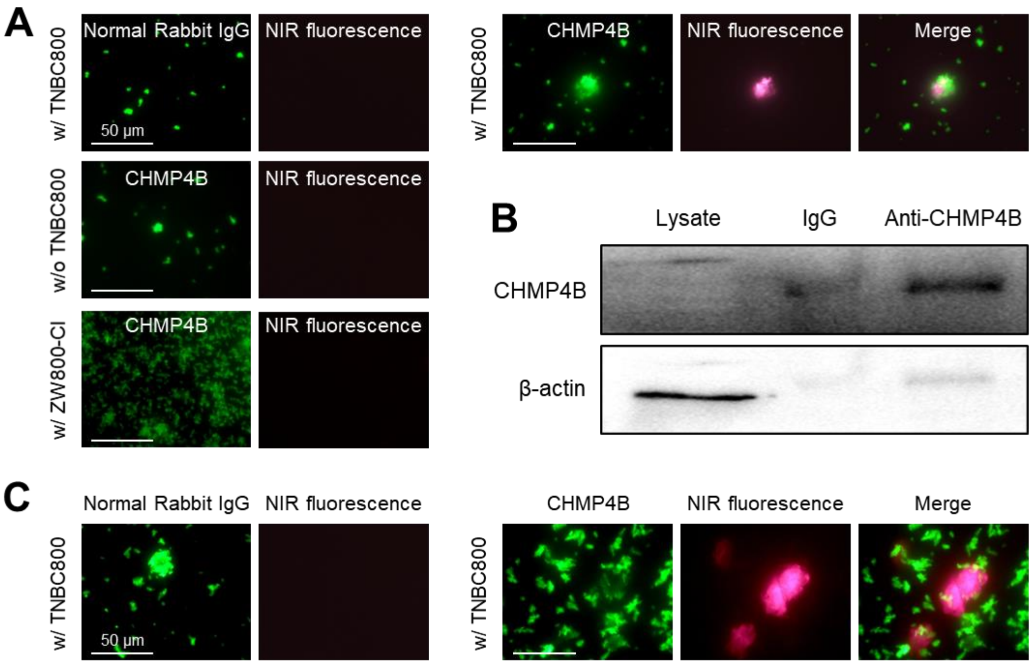

Figure S7. A) Immunoprecipitation assay of CHMP4B treated with ZW800-Cl or TNBC800 in MDA-MB-231 cells, followed by B) western blot analysis. C) Immunoprecipitation assay of CHMP4B treated with TNBC800 in MDA-MB-231 xenografted tumors.

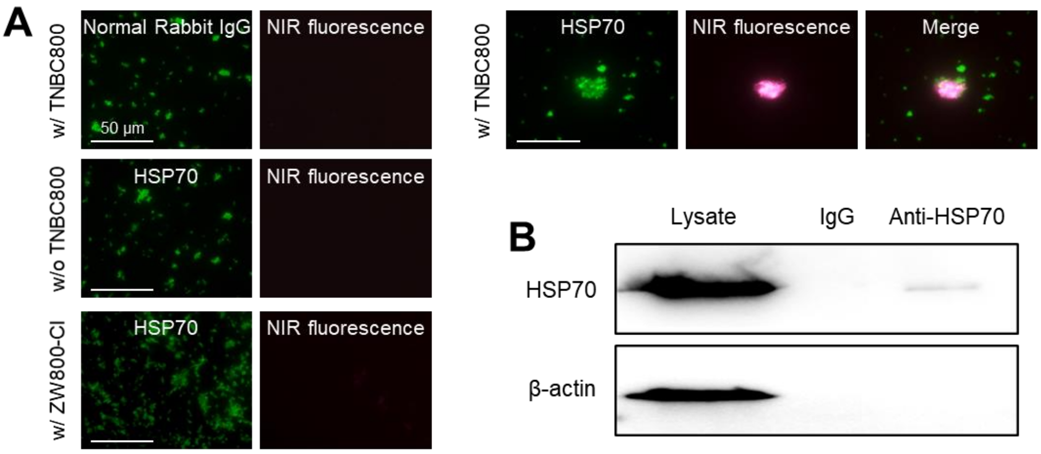

Figure S8. A) Immunoprecipitation assay of HSP70 treated with ZW800-Cl or TNBC800 in MDA-MB-231 cells, followed by B) western blot analysis.

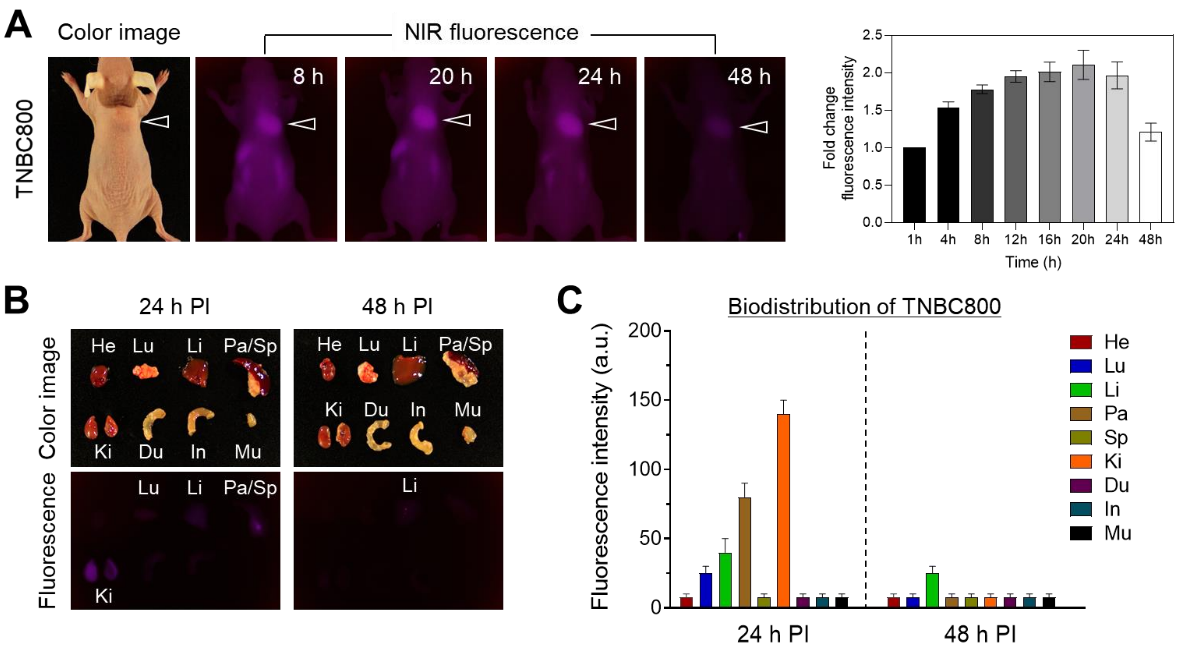

Figure S9. A) Time-dependent NIR fluorescence imaging and quantification for 48 h after injection of TNBC800. MDA-MB-231 tumor-bearing mice were intravenously injected with TNBC800 (1.0 mg kg^{-1}, $n = 3$). The tumor site is indicated by an arrowhead. B) Resected major organs imaged 24 and 48 h after injection of TNBC800. Images are representative of $n = 3$ independent experiments. All NIR fluorescence images had identical exposure times and normalization. C) Quantitative fluorescence analysis of intraoperative dissected organs imaged in B). Abbreviations: Du, duodenum; He, heart; In, intestine; Ki, kidneys; Li, liver; Lu, lungs; Mu, muscle; Pa, pancreas; Sp, spleen; and PI, post-injection.

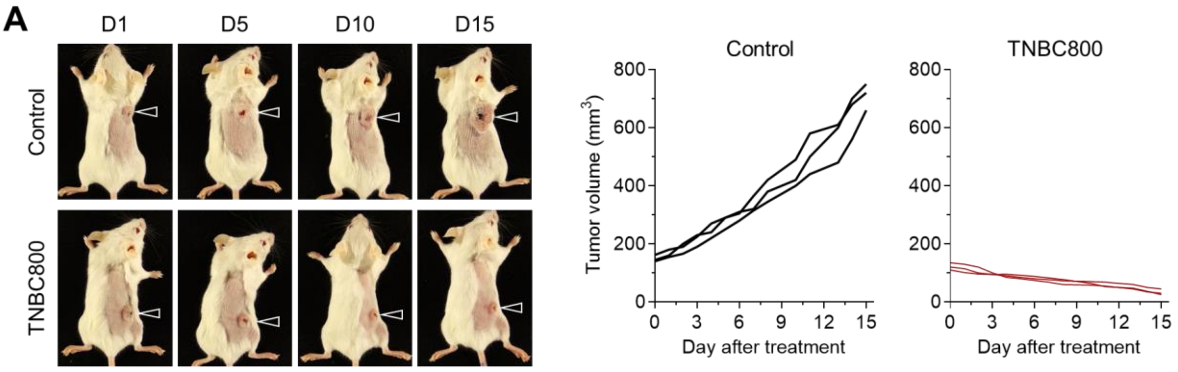

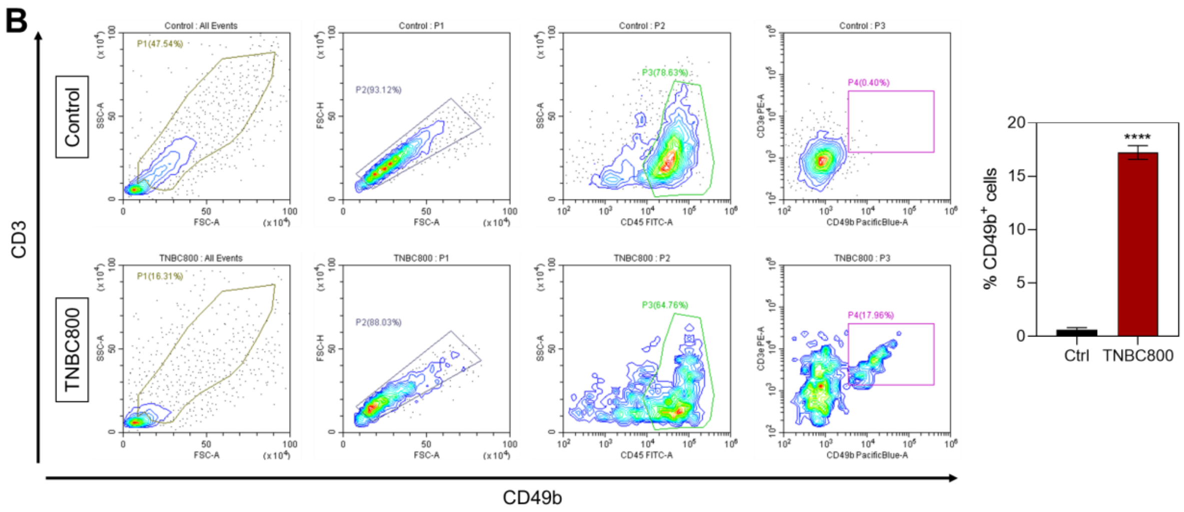

Figure S10. A) Antitumor effect of TNBC800 in the 4T1 xenograft mouse model. Tumor-bearing BALB/c mice were intravenously injected with TNBC800 (1.0 mg kg^{-1}, $n = 3$) every 2 days (a total of 5 times in 8 days). The tumor site is indicated by an arrowhead. Tumor growth rates of each treatment group were monitored for 15 days. B) Flow cytometry assay and quantification of NK cells (CD49b^{+}) in the tumor measured at day 3 after the 2nd treatment with TNBC800. Data are expressed as mean ± S.D. ($^{****}p < 0.0001$, $n = 3$).

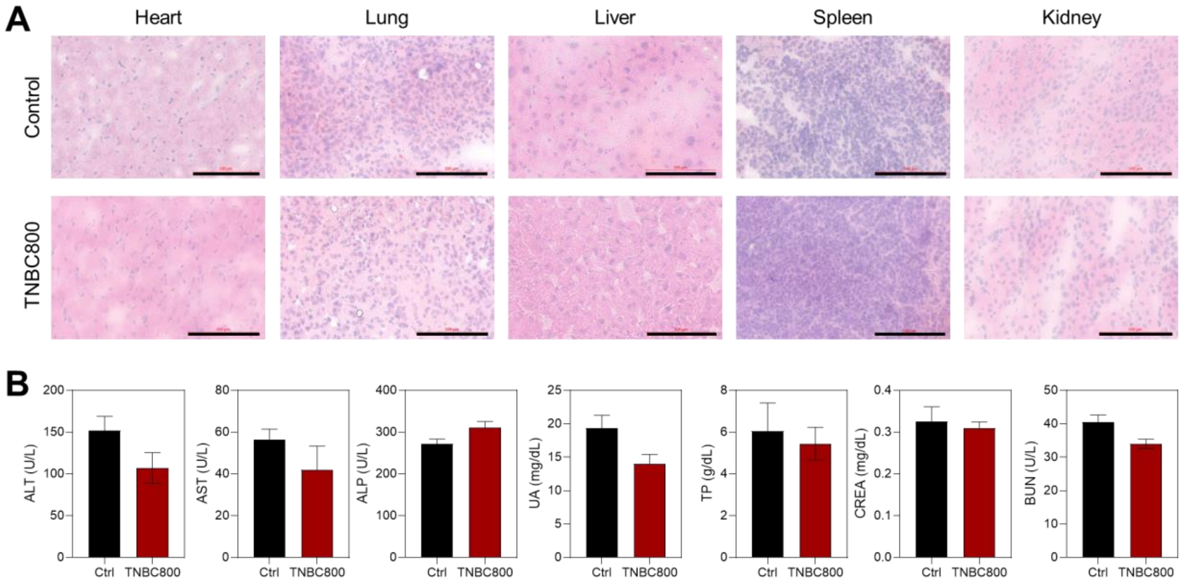

Figure S11. Biosafety evaluation of TNBC800. A) H&E stained images of major organs including heart, lung, liver, spleen, and kidney tissues harvested from each treatment group at day 15. Scale bars = 100 μm. B) Blood biochemical analysis at day 15 after intravenous injections of PBS and TNBC800, respectively.

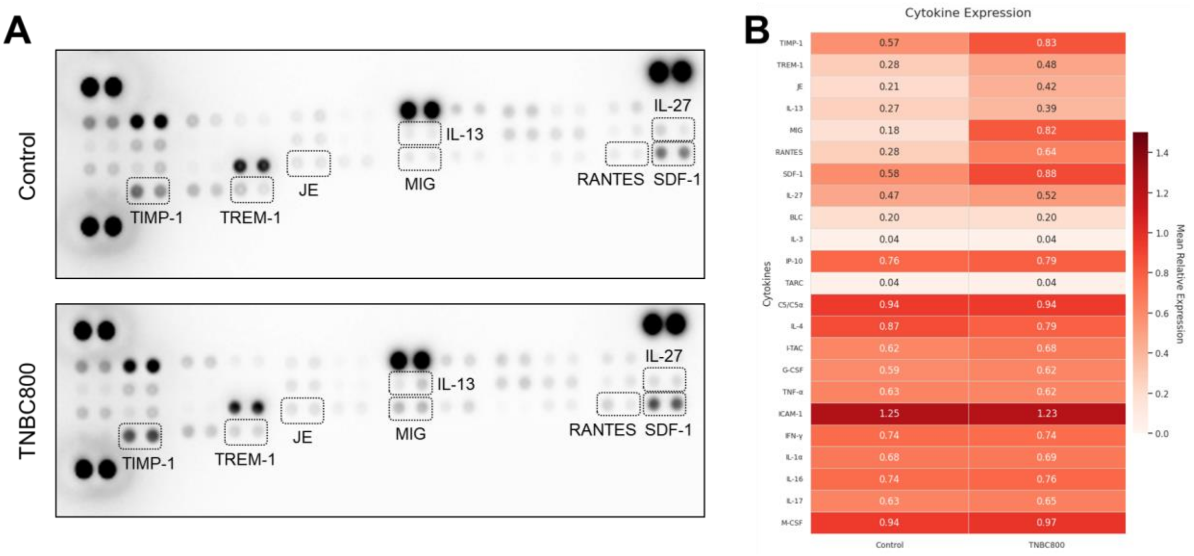

Figure S12. A) Cytokine profile and B) quantitative analysis of TNBC800. Each cytokine was detected in duplicate. In each array, blots in the upper-left, upper-right and bottom-left represent positive controls. Target cytokines are indicated with black square dotted lines.

Sample No. 1

Match Analysis

Locus	Reference Database Profile		Sample Profile		Shared alleles #
	Database : CRM-HTB-26		Sample Name : MDA-MB-231		
**D5S818**	12		12		1
**D13S317**	13		13		1
**D7S820**	8	9	8	9	2
**D16S539**	12		12		1
**vWA**	15	18	15	18	2
**TH01**	7	9.3	7	9.3	2
**TPOX**	8	9	8	9	2
**CSF1PO**	12	13	12	13	2
**AMEL**	X		X		1
**D3S1358**			16		-
**D21S11**			30	33.2	-
**D18S51**			11	16	-
**D8S1179**			13		-
**FGA**			22	23	-
**D2S1338**			20	21	-
**D19S433**			11	14	-
**Penta D**			11	14	-
**Penta E**			11		-
Number of shared alleles					14
Total number of alleles in the reference database profile					14
% match					100.0%
**Result interpretation**					**Related**

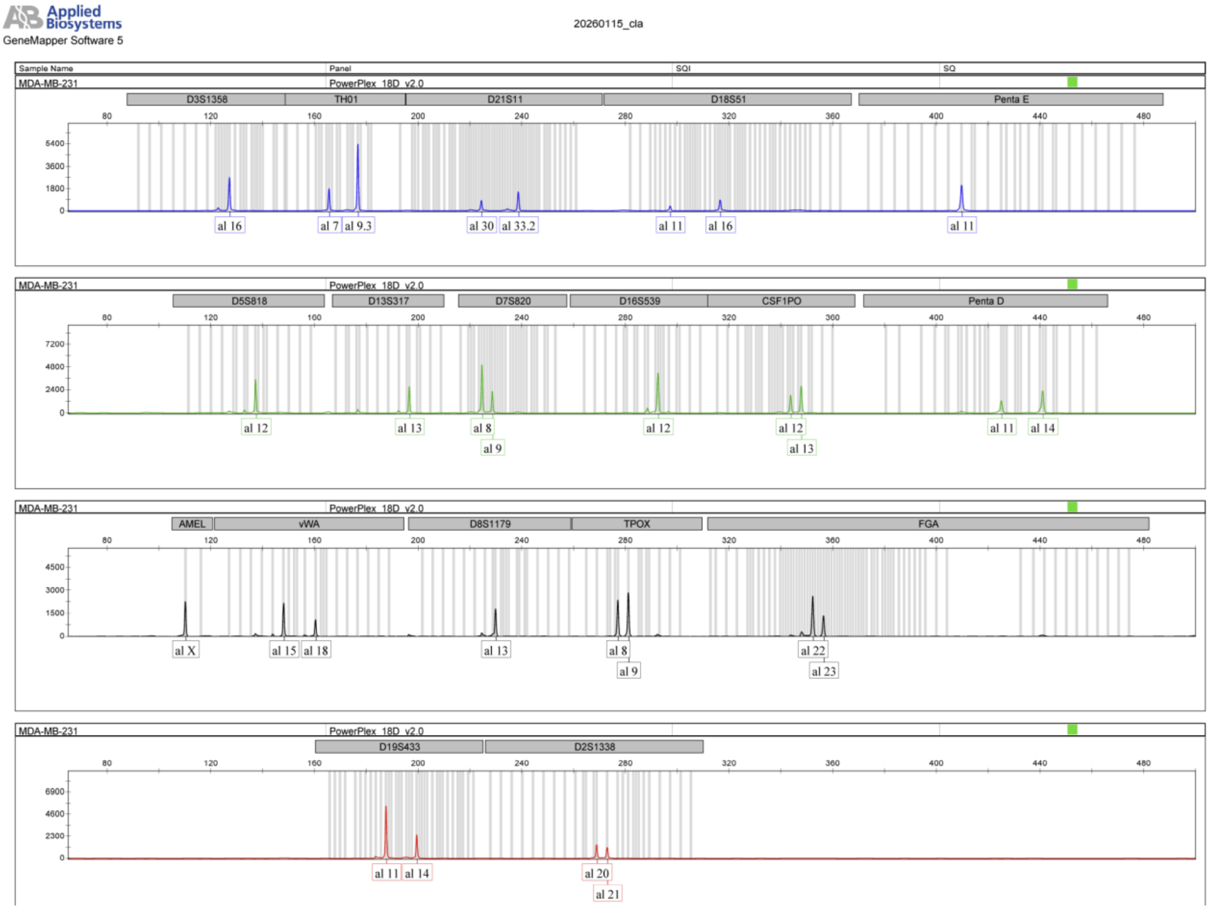

Figure S13. Short tandem repeat (STR) profiling for MDA-MB-231 cells.

#	Product	Supplier	Location	Lot number
1	Reagents and solvents	Sigma-Aldrich	St. Louis, MO, USA	
2	MDA-MB-231	ATCC	Manassas, VA, USA	HTB-26
3	MCF-7	ATCC	Manassas, VA, USA	HTB-22
4	4T1	ATCC	Manassas, VA, USA	CRL-2539
5	NIH/3T3	ATCC	Manassas, VA, USA	CRL-1658
6	RPMI 1640 medium	Welgene	Daegu, South Korea	
7	FBS	Welgene	Daegu, South Korea	
8	Antibiotics	Welgene	Daegu, South Korea	
9	Golgi-Tracker	APExBIO	Houston, TX, USA	B8813
10	Mito-Tracker	Thermo Fisher Scientific	Waltham, MA, USA	M7514
11	Lyso-Tracker	Thermo Fisher Scientific	Waltham, MA, USA	L7526
12	CHMP4B-targeting siRNAs	BIONEER	Daejeon, South Korea	
13	Lipofectamine^{TM} 3000	Thermo Fisher Scientific	Waltham, MA, USA	L3000001
14	TRIzol reagent	Sigma-Aldrich	St. Louis, MO, USA	T9424
15				

16	RevertAid Reverse Transcriptase	Thermo Fisher Scientific	Waltham, MA, USA	EP0441
17	Random hexamer primers	Thermo Fisher Scientific	Waltham, MA, USA	SO142
18	nTaq (Mg2+Plus) DNA polymerase	Enzynomics	Daejeon, South Korea	P025A
19	anti-CHMP4B antibody	Cell Signaling	Beverly, MA, USA	42466S
20	anti-HSP70 antibody	Invitrogen	Waltham, MA, USA	PA5-28003
21	Normal Rabbit IgG antibody	Cell Signaling	Beverly, MA, USA	2729S
22	Protein A/G magnetic beads	Thermo Fisher Scientific	Waltham, MA, USA	88802
23	Low pH elution buffer	Thermo Fisher Scientific	Waltham, MA, USA	21028
24	Neutralization buffer	Thermo Fisher Scientific	Waltham, MA, USA	15567027
25	BALB/c and female athymic nude mice	Orient Bio	Gwangju, South Korea	
26	Mouse Cytokine Array Kit	R&D Systems	Minneapolis, MN, USA	ARY006
27	TUNEL reagent	Promega	Madison, WI, USA	G3250
28	RIPA buffer	Biosesang	Yongin, South Korea	RC20020-050-00
29	Laemmli sample buffer	Bio-Rad	Hercules, CA, USA	1610737
30	PVDF membranes	Merck Millipore	Burlington, MA, USA	IPVH09120
31	BSA	Sigma-Aldrich	St. Louis, MO, USA	A9418
32	c-Caspase-3 antibody	Cell Signaling	Beverly, MA, USA	9661S
33	HSP70 antibody	Cell Signaling	Beverly, MA, USA	4876
34	LC3B antibody	Cell Signaling	Beverly, MA, USA	3868S
35	Bax antibody	Cell Signaling	Beverly, MA, USA	2772T
36	p-AKT antibody	Cell Signaling	Beverly, MA, USA	9271S
37	AKT antibody	Cell Signaling	Beverly, MA, USA	9272S
38	p-ERK antibody	Cell Signaling	Beverly, MA, USA	2234S
39	ERK antibody	Cell Signaling	Beverly, MA, USA	4348S
40	GAPDH	Santa Cruz	Dallas, TX, USA	sc-32233
41	β-actin	Santa Cruz	Dallas, TX, USA	sc-47778
42	anti-HMGB1 antibody	Cell Signaling	Beverly, MA, USA	3935
43	anti-calreticulin antibody	Cell Signaling	Beverly, MA, USA	12238
44	F4/80	BD Biosciences	San Jose, CA, USA	565411
45	MHC class II	Thermo Fisher Scientific	Waltham, MA, USA	11-5321-82
46	CD80	Thermo Fisher Scientific	Waltham, MA, USA	12-0801-82
47	CD49b	Thermo Fisher Scientific	Waltham, MA, USA	14-5971-85

Table S1. List of materials used in this study.

#	Instrument	Supplier	Location
1	Avance NMR spectrometer	Bruker	Billerica, MA, USA
2	Dionex UltiMate^{TM} 3000 mass spectrometer	Thermo Scientific	Waltham, MA, USA
3	FLAME spectrometer	Ocean Optics	Dunedin, FL, USA
4	SPARK^{TM} 10M instrument	Tecan	Männedorf, Switzerland
5	Fluorescence microscope	Nikon	Seoul, South Korea
6	FOBI fluorescence imaging system	NeoScience	Daejeon, South Korea
7	LuminoGraph III Lite imaging system	Atto	Tokyo, Japan
8	ZEISS LSM 900 confocal microscope	Carl Zeiss	Germany
9	CytoFLEX instrument	Beckman Coulter	Indianapolis, IN, USA

Table S2. List of instruments used in this study.
